# Relationship Between Homodimeric Glucocorticoid Receptor and Transcriptional Regulation Assessed via an *In Vitro* Fluorescence Correlation Spectroscopy-Microwell System

**DOI:** 10.1038/s41598-018-25393-w

**Published:** 2018-05-10

**Authors:** Sho Oasa, Shintaro Mikuni, Johtaro Yamamoto, Tsumugi Kurosaki, Daisuke Yamashita, Masataka Kinjo

**Affiliations:** 1 Laboratory of Molecular Cell Dynamics, Faculty of Advanced Life Science, Hokkaido University, Sapporo, 001-0021 Japan; 2 Laboratory of Molecular Cell Dynamics, Graduate School of Life Science, Hokkaido University, Sapporo, 001-0021 Japan

## Abstract

Glucocorticoid receptor (GR) is a hormone-activated transcription regulatory protein involved in metabolism as well as adrenocortical responses to psychosocial stress. Ligand-activated GR localizes to the nucleus, where GR homodimers regulate gene transcription via direct binding to glucocorticoid response elements (GREs). The role of GR homodimers in transcriptional activation has not yet been elucidated. In this study, we determined the concentration of GR homodimer, and its dissociation constant (*K*_d_), at the single-cell level, by using fluorescence correlation spectroscopy (FCS) combined with a microwell system. Results from dissociation constant analysis and diffusion analysis suggested that GR forms complexes with other proteins as well as homodimers. We determined the relationship between the concentration of GR homodimer and transcriptional activity using a triple-color FCS-microwell system-based fluorescent reporter assay. The binding affinity of GR to GREs was analyzed via fluorescence cross-correlation spectroscopy (FCCS). Our findings indicate that the GR homodimer is essential for activating target gene transcription.

## Introduction

Glucocorticoid receptor (GR) is a hormone-activated transcription regulatory protein and a member of the nuclear receptor superfamily, which is involved in modulating physiological processes and psychosocial stress responses^1–3^. Unactivated GR localizes to the cytoplasm, where it is complexed with chaperones and immunophilins^4,5^. After ligand binding, GR is translocated into the nucleus and subsequently forms GR homodimers, which then assemble into a protein complex with cofactors^6–8^. This complex regulates gene transcription via direct binding to the glucocorticoid response element (GRE)^9,10^. In addition, GR monomers and homodimers can tether other transcription factors, such as NF-κB, to suppress their activity^11,12^. Several alternative mechanisms for GR-dependent NF-κB repression have been suggested^13^. Prior studies identified GR homodimerization *in vitro* and *in vivo* and suggested that GR homodimerization is essential in regulating transcription^12,14–18^. Nevertheless, a correlation between monomeric/homodimeric state and transcriptional activity has not been established^12^. Recently, GR homotetramers on the DNA have been discovered^19,20^. It remains unclear which GR state is required to facilitate transcriptional activation.

**Figure 1:**
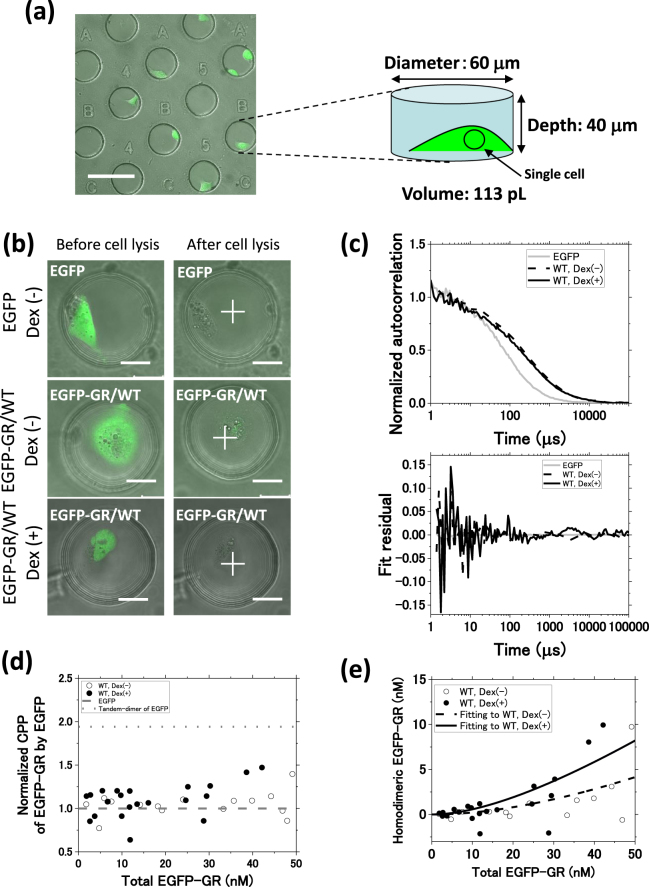
Homodimerization of wild-type glucocorticoid receptor assessed by an FCS-microwell system. (**a**) A fluorescent image of a single cell cultured in a microwell (diameter: 60 μm, depth: 40 μm, volume: 113 pL) on a polydimethylsiloxane (PDMS) chip. (**b**) A fluorescent image of a single cell expressing EGFP or EGFP-GR/WT, in the absence and presence of Dex, before and after cell lysis. White crosses indicate the position of FCS measurements. Scale bar: 20 μm. (**c**) Typical normalized autocorrelation functions and fit residuals for EGFP and EGFP-GR/WT in the absence and presence of Dex. Grey solid line: EGFP; black dashed line: EGFP-GR/WT in the absence of Dex; black solid line: EGFP-GR/WT in the presence of Dex. (**d**) Normalized CPP of EGFP-GR/WT in the 0–50 nM concentration range. The full concentration range is shown in Fig. S6A. Black open circle: GR/WT in the absence of Dex, black filled circle: GR/WT in the presence of Dex, grey dashed line: EGFP. (**e**) *K*_d,homo_ determination by non-linear least squares fitting of equation (16) (Supplemental Methods) in the 0–50 nM range. Black open circle: GR/WT in the absence of Dex (N = 21), black filled circle: GR/WT in the presence of Dex (N = 15), black dashed and solid lines: Fitting curve for GR/WT in the absence of Dex (*K*_d,homo_: 420 ± 120 nM, *Χ*^2^ = 4.9) and under Dex treatment (*K*_d,homo_: 140 ± 40 nM, *Χ*^2^ = 3.2). The *K*_d,homo_ values are averages ± SE. The full concentration range is shown in Fig. S6B. *Χ*^2^ represents the chi-square value per degree of freedom.

The GR homodimer may represent the basic state for binding to palindrome GRE and activating gene transcription. To investigate the relationship between GR homodimerization and transcriptional activity in individual cells, we used a single-cell method that combines fluorescence correlation spectroscopy (FCS) with microwell structures on polydimethylsiloxane (PDMS) chips (FCS-microwell system; Figs 1(a) and S1)^14,21^. FCS can determine both the particle number and diffusion time for fluorescently-tagged molecules in a sub-femtoliter detection volume^22^. These data can then be used to calculate the concentration and diffusion constant^14,23–25^. Moreover, a microwell on a PDMS chip provides a small cavity with stable conditions suitable for isolating proteins extracted from a single cell.

In a previous study, we determined the dissociation constant for GR homodimerization in HeLa cells by particle brightness analysis, using an FCS-microwell system^14^. Particle brightness is correlated to GR homodimer formation. To quantify the transcriptional activity of GR, a fluorescent reporter assay was carried out^26^. We therefore upgraded our FCS-microwell system to a triple-color FCS-microwell system (tcFCS-microwell system), in which EGFP-GR, mKO2 (fluorescent reporter for transcriptional activity), and TagRFP675 (transfection control) could be measured using FCS.

The DNA binding affinity of GR to GREs was determined using fluorescence cross-correlation spectroscopy (FCCS). FCCS is a widely-used technique for determining the interaction of molecules labeled with spectrally-distinct fluorophores^27,28^. Using FCCS data, dissociation constants of protein-protein interactions were determined *in vitro* and *in vivo*^18,29–32^.

In this study, we calculated dissociation constants for the homodimerization of wild-type GR and GR mutants in U2OS cells, using particle brightness analysis in an FCS-microwell system. The data obtained suggest that GR assembles into macromolecular complexes with endogenous interacting proteins. FCCS measurements and electrophoretic mobility shift assays also revealed that FCS-detected GR complexes can bind to GREs. Finally, the relationship between the concentration of GR homodimer and transcriptional activity in single cells was determined, using the tcFCS-microwell system. Our results indicate that GR homodimerization is a key contributor to transcriptional activation.

## Results

### Determination of GR homodimerization *in vitro*

We previously determined the dissociation constant for the homodimerization (*K*_d,homo_) of EGFP-fused wild-type GR (EGFP-GR/WT) in HeLa cells using the FCS-microwell system^14^. In the present study, we applied the FCS-microwell system to U2OS cells transfected with plasmids encoding EGFP or EGFP-GR/WT to determine the *K*_d,homo_. To confirm the endogenous expression of GR in these U2OS cells, western blotting was carried out (Fig. S2A). The large ratio of EGFP-GR expression to endogenous GR expression suggested that endogenous GR has little effect on the *K*_d,homo_ determination by heterodimer formation with endogenous GR.

As shown in Fig. 1(a), single cells were isolated into microwells (Diameter; 60 μm, Depth; 40 μm, Volume; 113 pL) on the PDMS chips. On the laser scanning microscopy (LSM) images, EGFP was distributed homogeneously in the living cell (Fig. 1(b), upper). In the absence of dexamethasone (Dex), EGFP-GR/WT localized in the cytoplasm (Fig. 1(b), middle), whereas it localized to the nucleus after the addition of Dex (Fig. 1(b), bottom). After cell lysis, the fluorescent signals of EGFP or EGFP-GR/WT in the cell decreased due to an extraction of EGFP or EGFP-GR/WT. To estimate an extraction efficiency of EGFP-GR/WT, the ratio of fluorescence intensity on the outside and inside of U2OS cells (CR_outside_/CR_inside_) was calculated (Fig. S3). This ratio reached a plateau (0.9) at 90 min following cell lysis, suggesting that almost all EGFP-GR is successfully extracted, as was observed in HeLa cells. Figure 1(c) shows typical autocorrelation functions and their fit residuals obtained from the FCS measurements in the microwells after cell lysis.

The counts per particle (CPP) was considered for fractions of homodimeric EGFP-GR; CPP was used as a proxy for homodimerization as the CPP of tandem-dimer of EGFP was twice as high as that of EGFP (Fig. S4). Figure 1(d) shows the CPPs of EGFP-GR/WT normalized by those of EGFP for 0–50 nM EGFP-GR/WT (full range graph in Fig. S6A). The normalized CPP of EGFP-GR/WT in the presence of Dex was higher than 1.0 and less than 2.0, which indicates that the EGFP-GR/WT was partially homodimerized. In contrast, in the absence of Dex, this parameter was measured consistently around 1.0 in the 0–50 nM range, and increased above 1.0 at higher concentrations of EGFP-GR/WT (Figs 1(d) and S6A). This result was consistent with a previous study, which identified GR homodimerization at high concentrations under uninduced conditions and monomeric GR at low concentrations^33^. The fraction of homodimeric EGFP-GR/WT was calculated from the normalized CPP (Fig. S5). Next, the concentration of homodimeric EGFP-GR/WT was calculated using the fraction of homodimeric EGFP-GR/WT and the concentration obtained from the in-microwell FCS measurement. The data were fitted to the bimolecular reaction model (equation (16) in Supplemental Methods) by using non-linear least squares curve fitting in the 0–50 nM concentration range, corresponding to the physiological concentration of GR (See Supplemental Note). Curve fitting using the entire observed concentration range yields higher chi-square values per degree of freedom (*Χ*^2^) compared with using only the 0–50 nM concentration range (Fig. S7). The *K*_d,homo_ of EGFP-GR/WT was determined to be 410 ± 120 nM (black dashed line) and 140 ± 40 nM (black solid line) in the absence and presence of Dex, respectively (Figs 1(e) and S6B). A comparable *K*_d,homo_ was calculated for EGFP-GR/WT in HeLa cells using the FCS-microwell system and in U2OS cells using the FCCS-microwell system^14,18^.

**Figure 2:**
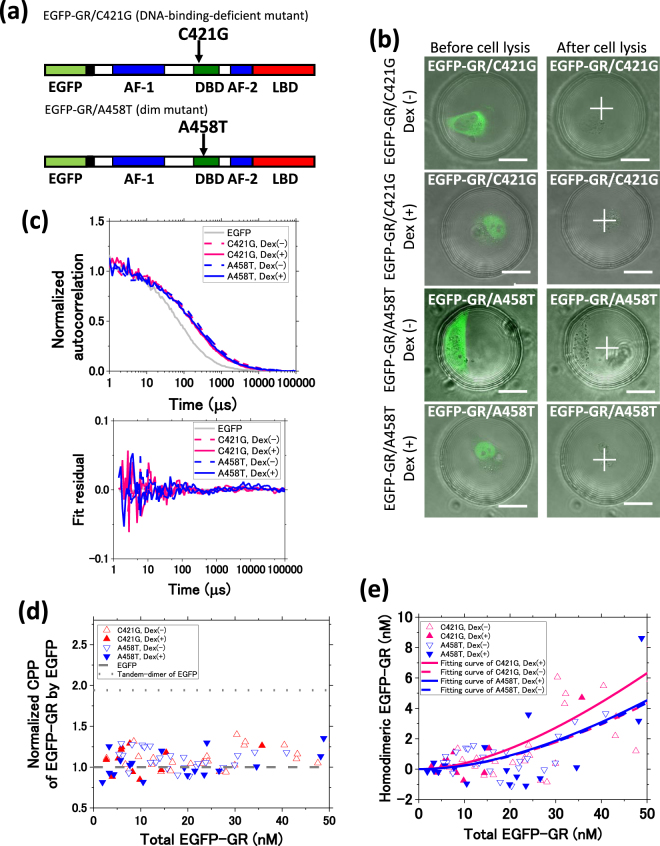
Homodimerization of glucocorticoid receptor mutants. (**a**) A schematic diagram of the EGFP-fused C421G mutant (DNA binding-deficient) and the A458T mutant (dim mutant for transcriptional activity). (**b**) Typical fluorescent images of a single cell expressing EGFP-GR/C421G or EGFP-GR/A458T, in the absence and presence of Dex, before and after cell lysis. White crosses indicate the position of FCS measurements. (Scale bar: 20 μm). (**c**) Typical normalized autocorrelation functions and fit residuals of EGFP-GR/C421G and EGFP-GR/A458T in the absence and presence of Dex. Grey solid line: EGFP, red dashed line: EGFP-GR/C421G in the absence of Dex, red solid line: EGFP-GR/C421G in the presence of Dex, blue dashed line: EGFP-GR/A458T in the absence of Dex, blue solid line: EGFP-GR/A458T in the presence of Dex. (**d**) The normalized CPP of EGFP-GR mutants in the 0–50 nM concentration range. The full concentration range is shown in Fig. S6C and D. Red open triangle: GR/C421G in the absence of Dex, red solid triangle: GR/C421G in the presence of Dex, blue open triangle: GR/A458T in the absence of Dex, blue solid triangle: GR/A458T in the presence of Dex, grey dashed line: EGFP. (**e**) *K*_d,homo_ determination by non-linear least squares fitting of equation (16) (Supplemental Methods) in the 0–50 nM range. Red open triangle: GR/C421G in the absence of Dex (N = 25); red solid triangle: GR/C421G in the presence of Dex (N = 11); blue open triangle: GR/A458T in the absence of Dex (N = 25); blue solid triangle: GR/A458T in the presence of Dex (N = 19); red dashed and solid lines: GR/C421G in the absence of Dex (*K*_d,homo_: 400 ± 100 nM, *Χ*^2^ = 0.5) and presence of Dex (*K*_d,homo_: 220 ± 30 nM, *Χ*^2^ = 2.7); blue dashed and solid lines: GR/A458T in the absence of Dex (*K*_d,homo_: 390 ± 80 nM, *Χ*^2^ = 0.7) and presence of Dex (*K*_d,homo_: 370 ± 120 nM, *Χ*^2^ = 2.6). The *K*_d,homo_ values are averages ± SE. The full concentration range is shown in Fig. S6E. *Χ*^2^ represents the chi-square value per degree of freedom.

Several studies have found that GR homodimerization occurred only on GRE^17,34,35^. However, others have shown that GR homodimerizes independently of DNA binding^18,36^. A458T mutant, commonly referred to as a dim mutant, was reported to suppress transcriptional activity and was unable to homodimerize; however, no direct result regarding GR homodimerization was shown^37^. To further investigate the relationship between GR homodimerization and DNA binding, U2OS cells were transfected with plasmids encoding two EGFP-GR mutants; C421G, a DNA binding-deficient mutant (Fig. 2(a), upper)^38^, and the A458T mutant (Fig. 2(a), bottom)^39^. The *K*_d,homo_ of EGFP-GR/C421G and EGFP-GR/A458T was compared with that of EGFP-GR/WT to reveal the effect of the DNA binding and A458T point mutation on GR homodimerization. Both mutants localized to the cytoplasm before Dex addition, but were found in the nucleus after Dex addition (Fig. 2(b)). Figure 2(c) shows typical autocorrelation functions and their fit residuals. The normalized CPPs of EGFP-GR/C421G and EGFP-GR/A458T were higher than 1.0 and less than 2.0 in the presence of Dex (Figs 2(d), S6C and S6D), suggesting that EGFP-GR/C421G and EGFP-GR/A458T were also partially homodimerized. The *K*_d,homo_ of EGFP-GR/C421G was determined to be 400 ± 100 nM (red dashed line) and 220 ± 30 nM (red solid line) in the absence and presence of Dex, respectively (Figs 2(e) and S6E), whereas the *K*_d, homo_ of EGFP-GR/A458T was calculated as 390 ± 80 nM (blue dashed line) in the absence of Dex and 370 ± 120 nM (blue solid line) with Dex addition (Figs 2(e) and S6E). Some data points at the higher total concentration of EGFP-GR show a greater homodimer concentration than expected from the fitted curve (Fig. S6B and E). This suggests the possible formation of higher-order oligomers, such as homotetramers, at this total concentration of GR.

**Figure 3:**
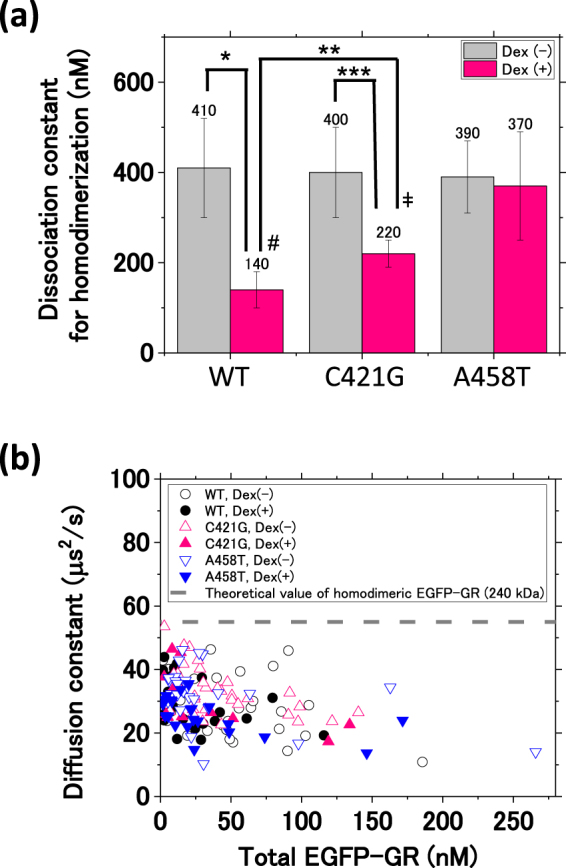
Summary of the dissociation constants of GR homodimerization and complex formation with endogenous interacting proteins. (**a**) A summary of the *K*_d,homo_ of GR/WT and mutants in the absence (grey bars) and presence (red bars) of Dex. GR/WT in the absence of Dex (*K*_d,homo_: 420 ± 120 nM) and presence of Dex (*K*_d,homo_: 140 ± 40 nM). GR/C421G in the absence of Dex (*K*_d,homo_: 400 ± 100 nM) and the presence of Dex (*K*_d,homo_: 220 ± 30 nM). GR/A458T in the absence of Dex (*K*_d,homo_: 390 ± 80 nM) and the presence of Dex (*K*_d,homo_: 370 ± 120 nM). Bars report averages ± SE. Statistical analysis was performed using Student’s *t*-test combined with the jackknife resampling method. *p = 7 × 10^−8^, **p = 0.003, ***p = 3 × 10^−6^, ^#^p = 4 × 10^−10^, ^ǂ^*p* = 7 × 10^−5^. (**b**) A summary of diffusion constants of wild-type GR and GR mutants in the absence and presence of Dex. Black open circle: GR/WT in the absence of Dex (N = 28); black solid circle: GR/WT in the presence of Dex (N = 25); red open triangle: GR/C421G in the absence of Dex (N = 35); red solid triangle: GR/C421G in the presence of Dex (N = 14); blue open triangle: GR/A458T in the absence of Dex (N = 28); blue solid triangle: GR/A458T in the presence of Dex (N = 21); grey dashed line: theoretical diffusion constant (55 μm^2^/s) calculated from the molecular weight of homodimeric EGFP-GR (240 kDa). Nearly all diffusion constants were between 10 and 47 μm^2^/s, which correspond to 470 kDa to 43 MDa.

Figure 3(a) summarizes the *K*_d,homo_ results. In the absence of Dex (gray bars), comparable *K*_d,homo_ values for EGFP-GR/WT and its mutants were calculated. The relatively high *K*_d,homo_ suggests that GR is homodimerized at a basal level, even when in the uninduced condition. EGFP-GR/A458T demonstrates the homodimerization in the absence and presence of Dex, with a relatively high *K*_d,homo_ as compared with EGFP-GR/WT, suggesting that the A458T mutant can homodimerize, but that this process is partially inhibited. In contrast, the *K*_d,homo_ of EGFP-GR/WT and EGFP-GR/C421G was significantly lower in the presence of Dex than that in its absence. These results indicate that the homodimerization of wild-type GR and the C421G mutant is induced by Dex and indicate that the DNA binding is not necessary for GR homodimerization. In the presence of Dex, the *K*_d,homo_ of EGFP-GR/C421G as compared to that of EGFP-GR/WT was significantly increased. This contrast suggests that the C421G point mutation may also affect GR homodimerization.

The diffusion constant of fluorescently-labeled proteins was quantified by FCS, and this value was affected by the complex size^40^. Figure 3(b) shows diffusion constants plotted against EGFP-GR total concentrations. The diffusion constants of EGFP-GR/WT and its mutants were lower than the dashed line, which was calculated from the molecular weight of homodimeric EGFP-GR (240 kDa). The diffusion constant of EGFP was 120 ± 3 μm^2^/s, which was the same as that of EGFP in the non-viscosity solution. Therefore, slow diffusion (a low diffusion constant) suggests high molecular weight complex formation by GR, in the absence as well as presence of Dex. The molecular weight of the GR complex was calculated to be in the range of 470 kDa to 43 MDa (10–47 μm^2^/s). These observations suggest that, in U2OS cells, wild-type GR and its mutants form not only homodimers, but also GR homotetramers (480 kDa) and macromolecular complexes with endogenous interacting proteins.

### Positive relationship between GR homodimerization and transcriptional activity

**Figure 4:**
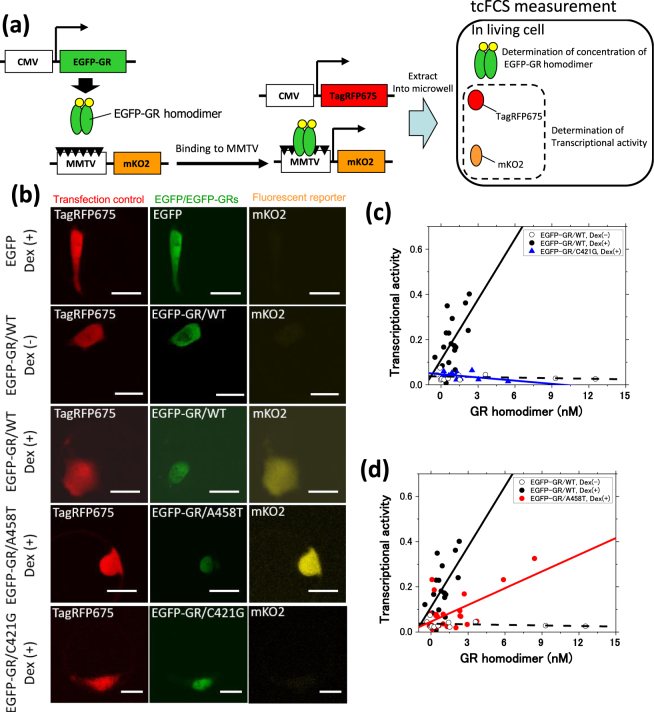
Positive relationship between the concentration of GR homodimer and transcriptional activity, determined using a tcFCS-microwell system. (**a**) A schematic diagram of triple-color FCS (tcFCS) measurement for a transcriptional activity assay. EGFP-GR binding to GREs (black triangle) in the MMTV sequence induces the expression of mKO2. After the extraction of EGFP-GR, mKO2, and TagRFP675 into the microwell, tcFCS measurement was performed. The transcriptional activity was calculated using the concentrations of mKO2 and TagRFP675 (transfection control) from tcFCS measurement. (**b**) Typical fluorescent images of EGFP or EGFP-GRs, mKO2, and TagRFP675 in the absence and presence of Dex. (Scale bar: 20 μm). **(c** and **d)** Transcriptional activity plotted against the concentration of GR homodimer in a single cell. Linear least squares fitting was carried out to determine the slope (Fig. S10) and the Pearson’s correlation coefficient (Fig. S11D). Black open circle: EGFP-GR/WT in the absence of Dex (N = 11), black solid circle: EGFP-GR/WT in the presence of Dex (N = 18), red circle: EGFP-GR/A458T in the presence of Dex (N = 24), blue triangle: EGFP-GR/C421G in the presence of Dex (N = 11), black dashed line: line of best fit of EGFP-GR/WT in the absence of Dex, black solid line: line of best fit of EGFP-GR/WT in the presence of Dex, red solid line: line of best fit of EGFP-GR/A458T in the presence of Dex, blue solid line: line of best fit of EGFP-GR/C421G in the presence of Dex.

We determined the relationship between GR homodimerization and transcriptional activity in each cell. To this end, a fluorescent reporter assay was integrated with the FCS-microwell system. The FCS-microwell system was upgraded to a triple-color FCS-microwell system (tcFCS-microwell system), which enabled us to measure the concentration of three spectrally distinct fluorescent proteins: EGFP or EGFP-GRs, mKO2 (fluorescent reporter for transcriptional activity), and TagRFP675 (transfection control), all extracted from a single cell. The fluorescent reporter comprised a mouse-mammalian tumor virus (MMTV) sequence as a GRE^41^, and mKO2 as a fluorescent reporter. The MMTV sequence that we used has four imperfect palindrome GREs (IpGREs) and two half palindrome GREs (hGREs)^42^. EGFP-GR binding to MMTV induced the expression of mKO2, after which the tcFCS-microwell system determined the concentrations of EGFP-GR for the GR homodimer, and mKO2 and TagRFP675 for the transcriptional activity (Fig. 4(a)). U2OS cells were co-transfected with plasmids encoding EGFP-GR, mKO2 (fluorescent reporter), and TagRFP675. The expression of EGFP-GR in the U2OS cells was very high compared to that of endogenous GR (Fig. S2B), suggesting that endogenous GR has a relatively minimal effect on the transcriptional activity induced by GR.

Figure 4(b) shows typical fluorescent images of EGFP or EGFP-GRs, mKO2 and TagRFP675. EGFP and TagRFP675 were distributed homogeneously in the living cells. EGFP-GR/WT localized mainly to the cytoplasm in the absence of Dex, while EGFP-GR/WT and its mutants localized to the nucleus after Dex addition (Figs 1(b), 2(b) and 4(b)). The fluorescent reporter signal of mKO2 was detected in the cells expressing EGFP-GR/WT and EGFP-GR/A458T after Dex addition. In contrast, the fluorescent signal of mKO2 was not detected in the cells expressing EGFP in the presence of Dex (N = 40), EGFP-GR/WT in the absence of Dex (N = 11), or EGFP-GR/C421G in the presence of Dex (N = 11) (Fig. 4(b)). These observations suggest that the expression of the fluorescent reporter (mKO2) was specifically induced by the direct binding of EGFP-GR to MMTV under Dex stimulation. Following cell lysis, typical autocorrelation functions and the fit residuals of EGFP or EGFP-GRs, mKO2, and TagRFP675 were obtained within the microwells (Fig. S8). These fits confirm a positive linear relationship between intracellular concentrations of mKO2 and TagRFP675 and their expression via a constitutive promoter (Fig. S9); this relationship suggests that TagRFP675 can be used to monitor relative amounts of transfected plasmids for the transcriptional activity. Transcriptional activity in each individual cell was calculated by dividing the concentration of mKO2 by that of TagRFP675. Figure 4(c) and (d) show the relationship between the concentration of GR homodimer and transcriptional activity. The transcriptional activity of EGFP-GR/WT in the presence of Dex (black filled circles) increased with the concentration of GR homodimer, whereas that of EGFP-GR/WT in the absence of Dex (black open circles) was consistently low. EGFP-GR/C421G transcriptional activity under Dex stimulation (blue filled down-triangle) was similar in tendency to that of EGFP-GR/WT without Dex exposure (Fig. 4(c)). These results suggest that GR homodimerization and its binding to the MMTV sequence in the nucleus are key factors in transcriptional activation. Interestingly, activation of transcription by EGFP-GR/A458T under Dex stimulation (red filled triangle) also increased with the concentration of GR homodimer (Fig. 4(d)), suggesting that, after Dex addition, the unstable A458T homodimer in the nucleus (*K*_d,homo_ = 370 nM; Fig. 3(a)) become able to activate transcription.

To reveal the relationship between the concentration of GR homodimer and transcriptional activity, we performed linear least squares fitting (Fig. 4(c) and (d)). Figure S10 shows the resulting slopes. Slopes representing EGFP-GR/WT and EGFP-GR/A458T in the presence of Dex differed significantly from those showing EGFP-GR/WT without Dex treatment and EGFP-GR/C421G under Dex stimulation. Moreover, in the presence of Dex, the slope of EGFP-GR/WT differed significantly from that of EGFP-GR/A458T, suggesting that the transcriptional activity of EGFP-GR/WT differed from that of EGFP-GR/A458T.

To further characterize the extent of the relationship between the concentration of GR homodimer and transcriptional activity, we determined Pearson’s correlation coefficient for the linear least squares fits (Fig. S11D). The Pearson’s correlation coefficients of EGFP-GR/WT and EGFP-GR/A458T in the presence of Dex are 0.59 and 0.83, respectively (red bar, Fig. S11D). We also utilized the tcFCS-microwell system to determine the concentration of GR monomer, total GR expression, fraction of GR homodimer, and concentration of GR homodimer. The linear least square fits determined the Pearson’s correlation coefficients for transcriptional activity and the concentration of GR monomer, total GR expression, and fraction of GR homodimer (Fig. S11A,B and C). For both EGFP-GR/WT and EGFP-GR/A458T, the highest correlation coefficients were those for transcriptional activity and the concentration of GR homodimer (Fig. S11D). These results strongly indicate a positive relationship between the concentration of GR homodimer and transcriptional activity in the presence of Dex, but no such relationship in its absence or for the DNA binding-deficient mutant C421G.

### Different DNA binding affinities of wild-type and A458T mutant to glucocorticoid response elements (GREs)

**Figure 5:**
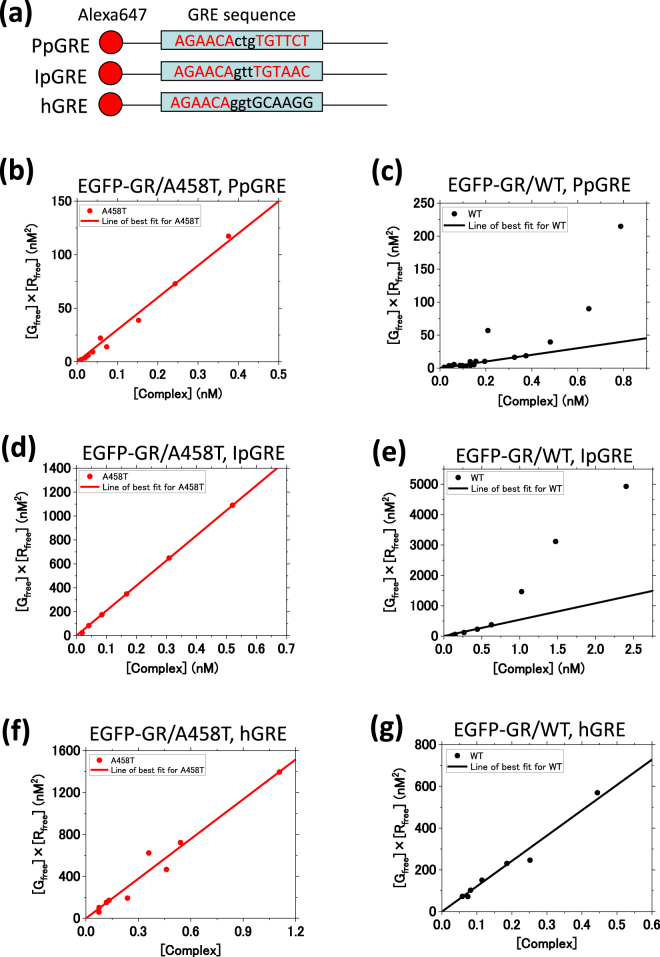
*In vitro* GR-DNA binding analysis using FCCS. (**a**) A schematic diagram of sequences of Alexa647-labeled GREs. PpGRE, IpGRE and hGRE denote perfect palindrome GRE (AGAACActgTGTTCT), imperfect palindrome GRE (AGAACAgttTGTAAC) and half GRE (AGAACAggtGCAAGG), respectively. IpGRE and hGRE were originated from the sequences in MMTV. (**b** and **c**) *K*_d,DNA,app_ determination for the interaction of EGFP-GRs and Alexa647-PpGRE, by a linear least squares fitting. **(b)** EGFP-GR/A458T (N = 14, *K*_d,DNA,app_: 300 ± 10 nM), **(c)** EGFP-GR/WT (N = 23, *K*_d,DNA,app_: 50 ± 3 nM). (**d** and **e**) *K*_d,DNA,app_ determination for the interaction of EGFP-GRs and Alexa647-IpGRE, by a linear least squares fitting. **(d)** EGFP-GR/A458T (N = 6, *K*_d,DNA,app_: 2100 ± 10 nM), **(e)** EGFP-GR/WT (N = 8, *K*_d,DNA,app_: 540 ± 40 nM). (**f** and **g**) *K*_d,DNA,app_ determination for the interaction of EGFP-GRs and Alexa647-hGRE, by a linear least squares fitting. **(f)** EGFP-GR/A458T (N = 9, *K*_d,DNA,app_: 1260 ± 60 nM), **(g)** EGFP-GR/WT (N = 7, *K*_d,DNA,app_: 1210 ± 50 nM).

To further reveal the elevated transcriptional activity of EGFP-GR/WT, relative to that of EGFP-GR/A458T, we determined the dissociation constant of binding between EGFP-GRs and GREs using fluorescence cross-correlation spectroscopy (FCCS) in a mixture of EGFP-GR lysates from U2OS cells and Alexa647-labeled DNAs (Alexa647-GREs). Figure 5(a) shows a schematic diagram of the Alexa647-GREs. Three types of GRE were used: PpGRE; Perfect palindrome GRE, IpGRE; Imperfect palindrome GRE, and hGRE; half palindrome GRE. IpGRE and hGRE originated from the MMTV sequence used.

The cross-correlation functions of EGFP, EGFP-GR/WT, and its mutants against Alexa647-GREs were normalized by an autocorrelation amplitude of EGFP or EGFP-GRs (Fig. S12). The amplitudes of normalized cross-correlation functions of EGFP-GR/WT (black solid line) and EGFP-GR/A458T (red solid line) were clearly observed, compared with those of EGFP (gray solid line) and EGFP-GR/C421G (blue solid line) (Fig. S12A,B and C), suggesting that the binding of EGFP-GR/WT and EGFP-GR/A458T to Alexa647-GREs was detected. To determine an apparent dissociation constant for the DNA binding of GRs (*K*_d,DNA,app_), concentrations of both bound [Complex] and free EGFP-GRs and Alexa647-GREs (EGFP-GR; [G_free_], Alexa647-GREs; [R_free_]) were calculated from the amplitudes of cross-correlation and autocorrelation functions, respectively^29^. Linear least squares fitting was applied to scatter plots of the product of the concentrations of free molecules [G_free_] × [R_free_] versus the concentration of the bound molecules [Complex]. The data were well fitted for EGFP-GR/A458T in the PpGRE, IpGRE and hGRE (*K*_d,DNA,app_ for PpGRE (Fig. 5(b)); 300 ± 10 nM, IpGRE (Fig. 5(d)); 2100 ± 10 nM, hGRE (Fig. 5(f)); 1260 ± 60 nM), and in the hGRE for EGFP-GR/WT (*K*_d,DNA,app_ for hGRE (Fig. 5(g)); 1210 ± 50 nM) in the presence of Dex, suggesting a single binding state such as monomer binding. In contrast, EGFP-GR/WT was not fitted in the PpGRE (Figs 5(c) and S13A, red solid line). This result suggests that GR/WT binds to PpGRE with multiple binding states. A similar trend was observed in the IpGRE for EGFP-GR/WT (Figs 5(e) and S13B, red solid line). To create a DNA binding model for WT, a simulation was carried out using two simple models incorporating monomer- and homodimer-binding to palindrome GRE in the absence and presence of homodimerization on the GREs (Fig. S14). Prior research has suggested a model in which DNA binding of the homodimer is stronger than that of the monomer when using purified GR^16^. Unexpectedly, our simulation results suggested that DNA binding of the monomer is stronger than that of the homodimer, based on FCCS data, as shown in Fig. 5(c) and (e). To calculate the *K*_d,DNA,app_ for monomeric DNA binding, linear least squares fitting to the data was performed. Three data points from the high complex concentration were excluded, due to chi-square value per degree of freedom (Fig. S13C). The *K*_d,DNA,app_ was determined to be 50 ± 3 nM and 540 ± 40 nM in the PpGRE and IpGRE for EGFP-GR/WT, respectively (Fig. 5(c) and (e)). These results suggest that monomeric EGFP-GR/WT binds strongly to PpGRE and IpGRE (*K*_d,DNA,app_ for PpGRE; 50 nM, IpGRE; 540 nM) whereas its homodimer exhibits only weak binding. In the PpGRE and IpGRE, the *K*_d,DNA,app_ value of monomeric EGFP-GR/WT was significantly lower than that of EGFP-GR/A458T (Student’s *t*-test between WT and A458T in PpGRE and IpGRE: *p* < 0.001). These results suggest that the monomeric DNA binding of WT which is stronger than that of the A458T mutant, initiates homodimerization with another GR monomer and that the transcriptional activity differs between WT and the A458T mutant.

To support the binding of EGFP-GRs and Alexa647-GRE, an electrophoretic mobility shift assay was performed (Fig. S15). In the fluorescent image of Alexa647-PpGRE, two shifts (GRS1 and GRS2) of EGFP-GR/WT and EGFP-GR/A458T were observed in the top part of the gel (GRS1) or faintly in the gel (GRS2) in lanes 1 and 2, compared with EGFP and Alexa647-PpGRE only in lanes 3 and 4 (Fig. S15A). As per the fluorescence intensity of Alexa647, the intensity of GRS1 in EGFP-GR/WT and EGFP-GR/A458T was significantly increased compared with that of EGFP, but that of GRS2 in EGFP-GR/WT and EGFP-GR/A458T was not significantly increased (Fig. S15C). The fluorescence of EGFP-GR/WT and EGFP-GR/A458T was also observed in the top part of the gel (GRS1) in lanes 1, 2 and 5, suggesting that the preformed protein complex of EGFP-GR, which forms independently by DNA binding, binds to Alexa647-PpGRE. There is a possibility that the shift of EGFP-GRs in the top part of the gel (GRS1) results from EGFP-GR aggregates with Alexa647-PpGRE. To rule out this possibility, western blotting was performed after ultra-centrifugation (Fig. S16). The ultra-centrifugation separated soluble from insoluble (aggregate) protein fractions^43^. The soluble fractions of EGFP-GR/WT and EGFP-GR/A458T were detected, but the insoluble fractions (aggregates) were not observed, suggesting that EGFP-GR/WT and EGFP-GR/A458T complexes with endogenous interacting proteins were functionally bound to Alexa647-PpGRE, but not as aggregates. The complex sizes of EGFP-GR and GREs were estimated from cross-correlation functions on FCCS data (Fig. S17). Almost all plots showed a size range from 250 kDa to 70 MDa, which was consistent with the range of 470 kDa to 43 MDa demonstrated using FCS data. This result suggests that GR homotetramers and large complexes with interacting proteins bind to PpGRE and are involved in regulating transcriptional activity.

## Discussion

In this study, we used an *in vitro* single-cell method combining FCS and microwells on a PDMS chip (FCS-microwell system). The FCS-microwell system was used to calculate the concentrations of homodimeric EGFP-GRs and their homodimerization dissociation constants. In addition, this method, combined with a fluorescent reporter assay enabled the determination of the positive relationship between the concentration of GR homodimer and transcriptional activity, in the presence of Dex.

The dissociation constant for the homodimerization of EGFP-GR/WT decreased under Dex treatment compared with untreated condition, which was consistent with our previous studies^14,18^. Furthermore, the dissociation constant of the DNA binding deficient C421G mutant also decreased with Dex stimulation; in contrast, the A458T mutant did not show such a reduction.

Prior studies on GR homodimerization have reported that the homodimerization of GR only occurrs on GREs in promoter regions^17,34,35^. We determined that the DNA binding deficient C421G mutant forms homodimers in the presence of Dex. This result indicates that DNA binding is not essential for GR homodimerization.

It has been reported that the A458T mutant can homodimerize when highly expressed^33^. We found that the homodimerization of the A458T mutant in the presence of Dex has a high dissociation constant, which was also observed for EGFP-GRs in the absence of Dex. These results suggest that, even without Dex stimulation, GR homodimer is formed in the cytoplasm at the basal level. Moreover, the homodimer of the A458T mutant exists in the nucleus after the addition of Dex, which suggests its involvement in transcriptional activity.

The diffusion constant obtained from FCS measurements indicated that GR forms a macromolecular complex with endogenous interacting proteins (470 kDa to 43 MDa; Fig. 3(b)). Previous studies identified interacting proteins, such as chaperones (Hsp40, Hsp70, Hsp90, and co-chaperones) and immunophilins (FKBP51 and FKBP52) in the cytoplasm^4,5^, and cofactors (e.g., GRIP1, NCOR1, MED14) in the nucleus^2^. These stable protein-protein interactions may regulate the transcription of genes via the coordination of GR homodimerization in living cells. To understand the effect of endogenous interacting proteins on GR homodimerization, it will be necessary to use mass spectroscopy to identify these proteins in the GR complex detected by FCS measurements.

The tcFCS-microwell system was used to determine the positive relationship between the concentration of the GR homodimer and transcriptional activity under Dex treatment. The Pearson’s correlation coefficient for the concentration of GR homodimer against transcriptional activity was higher than that calculated for GR monomer, the fraction of homodimer, as well as the total concentration (Fig. S11D). It was recently reported that the ratio of GR monomer and homodimer is not correlated with transcriptional activity^12^. Our results support this finding, as we observed a relatively low Pearson’s correlation coefficient for this measure (Fig. S11D). Pearson’s correlation coefficients reveal that the monomer concentration of GR (EGFP-GR/WT; 0.22, EGFP-GR/A458T; 0.42) and the total concentration of GR (expression level of GR) (EGFP-GR/WT; 0.18, EGFP-GR/A458T; 0.54) are less highly correlated with transcriptional activity than the GR homodimer. The relative tendency of Pearson’s correlation coefficients was similar between EGFP-GR/WT and the EGFP-GR/A458T mutant. Taken together, these results suggest that the concentration of GR homodimer in living cells is essential for regulating gene transcription.

**Figure 6:**
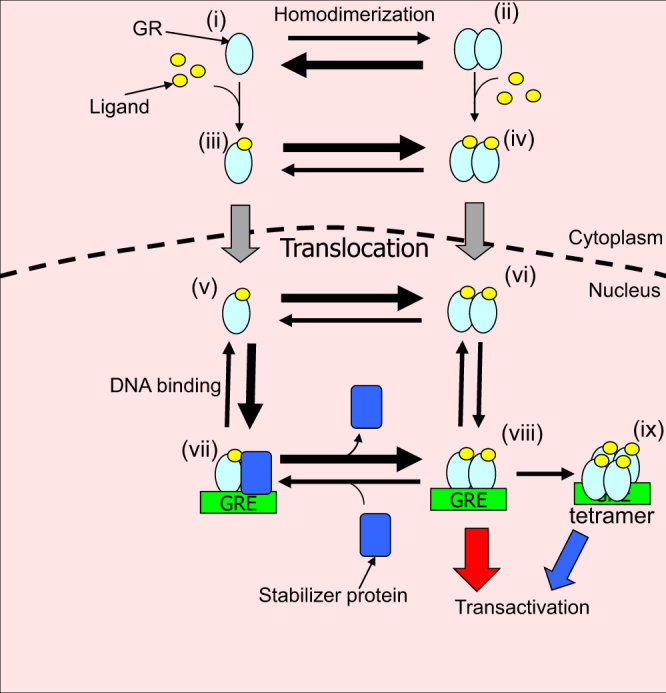
Schematic drawing of processes from GR homodimerization and transactivation. In the absence of ligand, GR in the cytoplasm forms a heterocomplex with chaperones, FKBP51, and other proteins (not shown in this figure). GR is homodimerized at a basal level ((i) ↔ (ii)). Upon ligand binding, GR tends to form homodimer during nuclear translocation ((iii) ↔ (iv)). After nuclear translocation ((iii) → (v), (iv) → (vi)), GR is able to homodimerize independently on the GRE ((v) ↔ (vi)). Monomer which may complex with stabilizer proteins binds strongly to GRE ((v) ↔ (vii)). Triggers such as exchange factors for stabilizer proteins, other monomers, and coactivators may convert stable monomer binding to homodimeric states on the DNA ((vii) ↔ (viii)). Transcriptional activity is altered based on the amount of homodimer, due to the increasing homodimer DNA binding fraction (red arrow). If the amount of GR homodimer increases, GR tetramerization, which prior research has shown^19,20^, takes place on the GRE ((viii) → (ix)) and may also be involved in modulating transcriptional activity (blue arrow).

The apparent dissociation constant for DNA binding of GRs revealed that the relationship between the concentration of GR homodimer and transcriptional activity differed between EGFP-GR/WT and EGFP-GR/A458T mutant (Figs 4(d) and 5). Previous work has suggested that the binding affinity of the GR homodimer to palindrome GRE is stronger than that of the monomer. In contrast, our FCCS and simulation data suggest that EGFP-GR/WT monomer binds strongly to palindrome GRE compared with its homodimer. This discrepancy may be caused by the presence of stabilizing proteins that interact with EGFP-GR monomer and contribute to strong monomeric DNA binding (Fig. 6). Data from the transcriptional activity assay support the notion that triggers such as exchange factors for stabilizer proteins, other GR monomers and coactivators may convert stable monomer binding to homodimeric states on the DNA to activate transcription in the nucleus. In contrast, data from the A458T mutant closely matched the line of best fit (Fig. 5(b),(d) and (f)), suggesting that this mutant binds to PpGRE, IpGRE, and hGRE as a monomer (Fig. S18B) with low binding affinity for GRE and with less binding propensity for stabilizer proteins, leading to the differing transcriptional activity observed for EGFP-GR/WT and EGFP-GR/A458T. Our results indicate that less than 30% of the A458T mutant formed the unstable homodimer (Fig. S11C). This small portion of unstable homodimer may be involved in the transcriptional activity of A458T mutant *via* direct binding to GRE. Single-molecule imaging has previously shown that the residence time of the A458T mutant on DNA is less than that of WT^44^. This suggests that unstable A458T homodimer has a shorter residence time than that of WT homodimer. Also, it has been reported that the dim mutation alters the allosteric effect that DNA exerts on GR, therefore varying the receptor conformations and perhaps changing its ability to interact with co-regulators^45^. Therefore, A458T mutant would be expected to demonstrate reduced transcriptional activity compared with that of WT.

Figure 6 summarizes the processes from GR homodimerization to transcriptional activity. Unliganded GR is homodimerized as a basal level (*K*_d,homo_; 400 nM, (i) ↔ (ii)). After the addition of Dex, the equilibrium tends towards the homodimer formation (*K*_d,homo_; 140 nM, (iii) ↔ (iv)) during nuclear translocation. After nuclear translocation, GR is homodimerized independently on DNA ((v) ↔ (vi)). The monomer complex with stabilizer protein binds strongly ((v) ↔ (vii)) compared with the homodimer ((vi) ↔ (viii)). In the living nucleus, triggers such as exchange factors for stabilizer proteins, another GR monomer, and coactivator recruitment can convert stable monomer binding to homodimer binding ((vii) ↔ (viii)). Transcriptional activity is correlated with the amount of homodimer, due to the increasing homodimer-DNA binding fraction in the living nucleus (red arrow). Recent work has suggested that GR homotetramerization on the DNA may occur^19,20^. The increase in the amount of GR homodimer may induce GR homotetramerization ((viii) → (ix)), and may also be involved in regulating transcriptional activity (blue arrow).

The electrophoretic mobility shift assay (Fig. S15) also demonstrates a shift in GR binding in the top part of the gel (GRS1) in the Alexa647-fluorescent image, as shown by fluorescence intensity (Fig. S15C). The molecular weight of the DNA-GR complex was calculated to be in the range of 250 kDa to 70 MDa (Fig. S17), which is consistent with the diffusion constant analysis (Fig. 3(b)). These results suggest that a macromolecular complex including GR binds to GRE and that the homotetramer and GR homodimer complexed with endogenous interacting proteins, such as cofactors, interact to activate gene transcriptions in living cells.

In conclusion, we used an FCS-microwell system to determine the dissociation constant for the homodimerization of GR, and complex formation of GR with endogenous interacting proteins. Moreover, the FCS-microwell system, combined with a fluorescent reporter assay, revealed the relationship between the concentration of GR homodimer and transcriptional activity in single cells. These results underscore the importance of GR homodimerization for transcriptional activation in living cells and contribute to our understanding of the molecular mechanisms underlying transcriptional regulation by nuclear receptors.

## Methods

### Chemicals and constructs

A synthetic ligand of GR, dexamethasone (Dex; Sigma-Aldrich, USA), was used at a concentration of 500 nM in Opti-MEM (Gibco Life Technologies, UK). The components of the lysis buffer were 80% CelLytic M Cell Lysis Reagent (Sigma-Aldrich, Israel) with PBS (pH 7.4), 1% Protease Inhibitor cocktail (Sigma-Aldrich, USA), 10 mM MgCl_2_, 0.1% SDS, and 200 U/mL Benzonase Nuclease (Sigma-Aldrich, USA). For the *in vitro* DNA binding assay and electrophoretic mobility shift assay, 500 nM Dex was added to lysis buffer, and SDS and Benzonase Nuclease were omitted.

The expression vectors for the enhanced green fluorescent protein (EGFP) and EGFP-fused wild type human glucocorticoid receptor α (EGFP-GR/WT) and its mutants (EGFP-GR/C421G and EGFP-GR/A458T) were described previously^23^. To determine the transcriptional activity, a vector encoding the mouse-mammalian tumor virus (MMTV) promoter region and orange fluorescent protein, mKO2, downstream of the MMTV promoter region (pMMTV-mKO2), was constructed. The MMTV sequence (RDB_05825) was from the RIKEN BioResource Center, and the mKO2 coding vector (phmKO2-MN1: AM-V0146) was purchased from MBL. The MMTV promoter region was amplified with PCR using a forward primer with AseI, and a reverse primer with NheI. The vectors encoding mKO2 and a PCR-amplified fragment of MMTV were digested by AseI and NheI sequentially. The linear vectors of mKO2 and digested MMTV were ligated by ligation mix (TaKaRa). pTagRFP675, which was purchased from Addgene (pTagRFP675-N1: Plasmid #44275) was co-transfected with pMMTV-mKO2 as a transfection control.

### Cell line and transfection

U2OS cells were purchased from ATCC (Lot# 58078676) and were maintained in McCoy’s 5 A modified medium (Gibco Life Technologies, UK) supplemented with 10% charcoal-stripped fetal bovine serum. FBS (Gibco Life Technologies, UK) was stripped overnight using 5% charcoal.

U2OS cells on the 6-well plate (Thermo Fisher Scientific) were transfected with 5 μg total plasmid DNA and ViaFect (Promega). For the determination of the dissociation constant for GR homodimerization, 5 μg pEGFP-GR/WT, its mutants, or 0.1 μg pEGFP and 4.9 μg empty vector were transfected. For elucidating the transcriptional activity in single cells, 1 μg pEGFP-GRs, 4 μg pMMTV-mKO2, and 0.1 μg pTagRFP675 were transfected. For *in vitro* DNA binding analysis by FCCS and electrophoretic mobility shift assay, U2OS cells on 10 cm dishes (Corning) were transfected with 15 μg pEGFP-GRs by ViaFect. At 6 h after the transfection, the cell culture medium was exchanged with fresh medium, and the U2OS cells were then cultured for 18 h.

To analyze single cells, individual transfected U2OS cells were seeded into microwells on the surface of polydimethylsiloxane (PDMS) chips (Fig. 1(a))^14^. The cell suspension with 3.0 × 10^4^ (cells/mL) was poured on the PDMS chips after washing with detergent water. Cells were incubated to adhere cells to PDMS chips for 4 h.

### Single-cell method using an FCS-microwell system

The PDMS chips with microwells were originally designed and ordered from Fluidware Technologies Inc., Tokyo, Japan. The microwells were 60 μm in diameter and 40 μm in depth, with a volume of 113 pL (Fig. 1(a), right). Each microwell was denoted by a number and a letter (Fig. 1(a), left). The PDMS chips and coverslips (No. S1: Matsunami Glass, Tokyo, Japan) were treated with N101 blocking reagent (Nippon Oil and Fats, Tokyo, Japan) to prevent protein adsorption.

Schematic diagrams of the FCS-microwell system are shown in Fig. S1. The microwell PDMS chip was attached to a glass stick with double-sided tape (Nitoms, Tokyo, Japan), pressed onto a coverslip in Opti-MEM with Dex, and incubated at 37 °C for 20 min to activate GRs (Fig. S1A). The medium on the coverslip was changed to lysis buffer, and the protein extracted from each single cell was retained in the microwell after cell lysis, after which FCS measurements were carried out in each microwell (Fig. S1B).

### LSM imaging and FCS/FCCS measurements

Laser scanning microscopy (LSM) imaging and FCS measurements were obtained using an LSM510-Confocor2 (Carl Zeiss) equipped with an Ar-ion laser, He-Ne lasers, a water immersion objective (C-Apochromat, 40×, 1.2 NA, Corr, Carl Zeiss, Germany), photomultiplier tubes (PMTs) for LSM imaging, and avalanche photodiode detectors (APDs) for FCS measurements.

For the determination of the dissociation constants using the FCS-microwell system, EGFP was excited using a 488 nm laser. The pinhole size was adjusted to 70 μm. The fluorescence passing though LP 505 was detected by PMT for LSM imaging, and the fluorescence passing though BP 505–550 was detected by APD for FCS measurement. FCS measurements were carried out five times for a duration of 10 s.

For the quantification of the concentration of GR homodimer and transcriptional activity using the tcFCS-microwell system, EGFP, mKO2, and TagRFP675 were excited with 488 nm, 543 nm and 633 nm lasers, respectively. In LSM imaging, the pinhole size was adjusted to 140 μm for EGFP, 1000 μm for mKO2, and 280 μm for TagRFP675. The fluorescence passing through BP500–530 for EGFP, BP565–615 for mKO2, and LP650 for TagRFP675 was detected by PMTs. For tcFCS measurement, the pinhole size was adjusted to 70 μm for EGFP, 78 μm for mKO2, and 90 μm for TagRFP675. The fluorescence passing through BP505–550 for EGFP, BP565–615, and LP650 was detected by APDs. To prevent cross-talk between channels, LSM imaging and FCS measurement were sequentially performed for EGFP/TagRFP675 and for mKO2. The fluorescence from EGFP and TagRFP675 was split by a dichroic mirror (635 nm). FCS measurement was performed 5 times for a duration of 10 s.

In multi-color imaging and tcFCS measurement, cross-talk in each detector is serious problem encountered when quantifying the fluorescent protein concentration. The cross-talk between channels was confirmed by tcFCS measurements with lysates containing each fluorescent protein. The counts per particle (CPP) from the FCS measurements are summarized in Table S1. If a high fluorescent protein CPP value was observed in the specific channel as well as in others, cross-talk occurred between the channels. Since high CPP values for each fluorescent protein were observed in each specific channel, this tcFCS measurement method could determine the number of particles of each fluorescent protein, even if these fluorescent proteins were mixed in the lysate.

For *in vitro* DNA binding analysis by FCCS, EGFP and Alexa647 were excited using 488 nm and 633 nm lasers, respectively. The fluorescence from EGFP and Alexa647 was split using a dichroic mirror (635 nm), following which the split fluorescence passed through a 70 μm pinhole and BP505–550 for EGFP, or a 90 μm pinhole and LP650 for Alexa647, and was then detected by APDs. The FCCS measurement was performed 5 times for a duration of 10 s.

### Transcriptional activity assay using the tcFCS-microwell system

Following transfection of U2OS cells with pEGFP-GR, pMMTV-mKO2, and pTagRFP675, cells were incubated for 24 h and treated with 500 nM Dex for 18 h to activate GR-mediated gene expression. During Dex treatment, U2OS cells were spread into a microwell chip with medium containing 500 nM Dex. After Dex treatment, single U2OS cells were lysed to extract EGFP-GR, mKO2, and TagRFP675 into the microwells, after which tcFCS measurement was carried out to determine their concentrations. Transcriptional activity was calculated by dividing the mKO2 concentration by the TagRFP675 concentration.

### Synthesis of Alexa647-labeled GRE for calculation of the *in vitro* dissociation constant of DNA binding by using FCCS measurement

The template containing GRE sequences was extended using an Alexa647-labeled common primer and a Klenow fragment (TaKaRa) at 37 °C for 30 min. After extension, exonuclease treatment was used to digest the unreacted single-strand DNA. Alexa647-labeled DNA was purified using an Illustra AutoSeq G-50 column (GE Healthcare). The whole sequence of the Alexa647-labeled GRE constructs is shown below. The capital letter show the GREs.

PpGRE (36 bp); 5′-Alexa647-tcgagggatccgaattcaAGAACActgTGTTCTctc-3′

IpGRE (40 bp); 5′-Alexa647-tcgagggatccgaattcAGAACAgttTGTAACcaaaaact-3′

hGRE (40 bp); 5′-Alexa647-tcgagggatccgaattcAGAACAgttgcaaggactattga-3′.

## Electronic supplementary material

Supplementary information
