# Supplementary material for: Relationship Between Homodimeric Glucocorticoid Receptor and Transcriptional Regulation Assessed via an In Vitro Fluorescence Correlation Spectroscopy-Microwell System
Source: Sci Rep. 2018 May 10;8:7488. doi: 10.1038/s41598-018-25393-w (PMC5945783; doi:10.1038/s41598-018-25393-w)
Supplement: Supplementary file 1 — Supplementary information [file 41598_2018_25393_MOESM1_ESM.pdf]

Relationship Between Homodimeric Glucocorticoid Receptor and Transcriptional Regulation Assessed via an *In Vitro* Fluorescence Correlation Spectroscopy-Microwell System

Sho Oasa[1], Shintaro Mikuni[1], Johtaro Yamamoto[1], Tsumugi Kurosaki[2], Daisuke Yamashita[2], and Masataka Kinjo[1,*]

1. Laboratory of Molecular Cell Dynamics, Faculty of Advanced Life Science, Hokkaido University, Sapporo 001-0021 Japan.
2. Laboratory of Molecular Cell Dynamics, Graduate School of Life Science, Hokkaido University, Sapporo 001-0021 Japan

* Correspondence: kinjo@sci.hokudai.ac.jp

Supplementary methods

Data analysis for FCS measurements

Data obtained from the FCS measurements were calculated using AIM software (Carl Zeiss). The autocorrelation function $G_i(\tau)$ was defined as follows:

$$G_i(\tau) = \frac{\langle I_i(t) I_i(t+\tau) \rangle}{\langle I_i(t) \rangle^2} \qquad \text{- (1)}$$

where *i* indicates EGFP-GR, mKO2, or TagRFP675; τ shows the delay time, and *I* is the fluorescence intensity. The obtained autocorrelation functions were fitted using a one-component model as follows:

$$G_i(\tau) = 1 + \left(1 + \frac{F_{i,\mathrm{triplet}}\, e^{-\frac{\tau}{\tau_{i,\mathrm{triplet}}}}}{1 - F_{i,\mathrm{triplet}}} \right) \cdot \frac{1}{N_i} \left(1 + \frac{\tau}{\tau_{i,D}} \right)^{-1} \cdot \left(1 + \frac{1}{s_i^2} \frac{\tau}{\tau_{i,D}} \right)^{-\frac{1}{2}} \qquad \text{- (2)}$$

where *i* indicates EGFP-GR, mKO2, or TagRFP675; F_{triplet} is the average fraction of the triplet state of fluorescent particles; τ_{triplet} is the average relaxation time of the triplet time; and τ_{D} is the average diffusion time of florescent particles. The diffusion constant of EGFP-GR was calculated from the diffusion constant of a standard molecule, rhodamine 6G (D_{R6G}; 414 μm^2/s)[1], and the ratio of diffusion times τ_{R6G} and τ_{D}. *N* is the average number of fluorescent particles in the effective observation volume ($V_{i,\,\mathrm{eff}}$) defined by 3D Gaussian volume elements with lateral radius $w_{i,0}$ and axial radius $z_{i,0}$. s_i

shows the structure parameter representing the ratio of $w_{i,0}$ to $z_{i,0}$ ($s_i = z_{i,0}/\ w_{i,0}$). $w_{i,0}$ and $z_{i,0}$ were determined by calibration measurement with R6G (D_{R6G}; 414 μm^2/s) for the EGFP and mKO2 channels, and Alexa647 ($D_{Alexa647}$; 330 μm^2/s)[2] for the TagRFP675 channel, respectively.

$$w_{i,0} = \sqrt{4D_i \cdot \tau_i} \qquad \text{- (3)}$$

$$s_i = \frac{z_{i,0}}{w_{i,0}} \qquad \text{- (4)}$$

The effective observation volume was calculated using the following equation:

$$V_{i,\,\mathrm{eff}} = \pi^{\frac{3}{2}} \cdot w_{i,0}^2 \cdot z_{i,0} \qquad \text{- (5)}$$

To remove the effect of background fluorescence on the measured N_{meas}, the background-corrected N (N_{corr}) was calculated as follows[3,4]:

$$N_{i,\mathrm{corr}} = \frac{N_{i,\mathrm{meas}} \cdot (I_{i,\mathrm{meas}} - I_{i,\mathrm{B}})^2}{(I_{i,\mathrm{meas}})^2} \qquad \text{- (6)}$$

where i indicates EGFP-GR, mKO2, and TagRFP675. $N_{i,\mathrm{meas}}$ is the number of fluorescent particles measured by FCS, $I_{i,\mathrm{meas}}$ is the measured average fluorescence intensity, and $I_{i,\mathrm{B}}$ is the average background fluorescence intensity from FCS measurement using a non-transfected U2OS cell lysate.

The concentration of fluorescent particles ([$C_{i,\mathrm{corr}}$]) was calculated from the

effective observation volume ($V_{i,\mathrm{eff}}$), the corrected number of fluorescent particles ($N_{i,\mathrm{corr}}$), and Avogadro's number ($\mathrm{N_A}$) as given below:

$$\left[C_{i,\mathrm{corr}}\right] = \frac{N_{i,\mathrm{corr}}}{V_{i,\mathrm{eff}} \cdot \mathrm{N_A}} \quad - (7)$$

Determination of the GR homodimer concentration and its dissociation constant by FCS

The calculation method used here was described in our previous work[3]. Briefly, the monomeric fraction F_m and the homodimeric fraction F_d (F_m+F_d =1) of EGFP-GR were calculated using equations (8), (9), and (10). The apparent particle brightness defined as counts per particle (CPP) was calculated by the division of fluorescence intensity by the number of fluorescent particles. If the monomeric and homodimeric GR are contained in the lysate, the apparent CPP ($CPP_{\text{EGFP-GR, app}}$) is obtained using the following equation:

$$CPP_{\text{EGFP-GR,app}} = \frac{F_\mathrm{m} \cdot \eta_\mathrm{m}^2 + F_\mathrm{d} \cdot \eta_\mathrm{d}^2}{F_\mathrm{m} \cdot \eta_\mathrm{m} + F_\mathrm{d} \cdot \eta_\mathrm{d}} \quad - (8)$$

where η_m and η_d are the CPP of monomeric and homodimeric EGFP-GR, respectively. Since the CPP of the tandem dimer of EGFP was twice as high as that of EGFP (Fig. S4), η_m and η_d can be defined using the CPP of EGFP (CPP_EGFP).

$$\eta_{m} = CPP_{EGFP} \quad \text{- (9)}$$

$$\eta_{d} = 2 \cdot CPP_{EGFP} \quad \text{- (10)}$$

Using equations (8), (9), and (10), the fractions of monomeric and homodimeric EGFP-GR are as shown below.

$$F_{m} = \frac{4 - 2R}{3 - R} \quad \text{- (11)}$$

$$F_{d} = \frac{R - 1}{3 - R} \quad \text{- (12)}$$

where

$$R = \frac{CPP_{EGFP\text{-}GR,app}}{CPP_{EGFP}} \quad \text{- (13)}$$

The concentrations of monomeric and homodimeric GR, [M] and [D] respectively, were calculated using each fraction and the concentration from the FCS measurement in equation (7), as follows:

$$[\mathrm{M}] = F_{m} \times [C_{EGFP-GR,\ corr}] \quad \text{- (14)}$$

$$[\mathrm{D}] = F_{d} \times [C_{EGFP-GR,\ corr}] \quad \text{- (15)}$$

To determine the dissociation constant for the homodimerization of EGFP-GRs, non-linear least squares fitting was performed using equation (16).

$$[\mathrm{D}] = \frac{K_{d} + 4[\mathrm{M}_{0}] - \sqrt{(K_{d} + 4[\mathrm{M}_{0}])^{2} - 16[\mathrm{M}_{0}]^{2}}}{8} \quad \text{- (16)}$$

where

$$[M_0] = [M] + 2[D] \quad - (17)$$

In vitro DNA-binding analysis using FCCS measurement

After a 20-min stimulation with 500 nM Dex, the transfected U2OS cells on a 10 cm cell culture dish were washed with ice cold PBS (pH 7.4), trypsinized, and collected in PBS (pH 7.4) containing the trypsin inhibitor 4-[2-aminoethyl] benzenesulfonyl fluoride hydrochloride (Sigma-Aldrich, USA). The cell number was counted. The lysis buffer was calculated from the cell number and microwell volume (113 pL) to prepare the EGFP or EGFP-GRs lysate with the endogenous protein concentration equal to that of protein extracted from a single cell in a microwell. After centrifugation, the cell pellet was suspended in the lysis buffer. The cell lysates were sonicated on ice. The homogenates were centrifuged (17400 ×*g*, 4 °C) for 15 min, and the supernatant were collected. The total protein concentrations in EGFP, EGFP-GRs, or non-transfected cell lysates was measured by the Bradford method and adjusted to the same concentration in all samples. FCCS measurement was performed five times for 10 s for the mixture of EGFP-GRs and Alexa647-labeled GREs.

Data acquired from FCCS were analyzed using AIM software (Carl Zeiss). The

autocorrelation functions from green and red channels, $G_G(\tau)$ and $G_R(\tau)$ respectively, and the cross-correlation function $G_C(\tau)$ were calculated as follows:

$$G_i(\tau) = \frac{\langle I_i(t) I_i(t+\tau)\rangle}{\langle I_i(t)\rangle^2} \qquad - (18)$$

$$G_C(\tau) = \frac{\langle I_G(t) I_R(t+\tau)\rangle}{\langle I_G(t)\rangle \cdot \langle I_R(t)\rangle} \qquad - (19)$$

where i indicates the green or red channel, τ denotes the delay time, and I_i is the fluorescence intensity of the green or red channel, respectively. The obtained auto- and cross-correlation functions were fitted using a one-component model as follows:

$$G_{i,\,\mathrm{auto}}(\tau) = 1 + \left(1 + \frac{F_{\mathrm{triplet}}\, e^{-\frac{\tau}{\tau_{\mathrm{triplet}}}}}{1 - F_{\mathrm{triplet}}}\right) \cdot \frac{1}{N}\left(1 + \frac{\tau}{\tau_D}\right)^{-1} \cdot \left(1 + \frac{1}{s^2}\frac{\tau}{\tau_D}\right)^{-\frac{1}{2}} \qquad - (20)$$

$$G_{\mathrm{cross}}(\tau) = 1 + \frac{1}{N}\left(1 + \frac{\tau}{\tau_D}\right)^{-1} \cdot \left(1 + \frac{1}{s^2}\frac{\tau}{\tau_D}\right)^{-\frac{1}{2}} \qquad - (21)$$

where i indicates the green or red channel, F_{triplet} is the average fraction of the triple state of fluorescent particles, τ_{triplet} is the average relaxation time of the triplet state, and τ_D is the average diffusion time of fluorescent particles. N is the average number of fluorescent particles in the effective observation volume, and s is the structure parameter ($s_i = z_{i,0}/w_{i,0}$). Values of the lateral radius $w_{i,0}$ and axial radius $z_{i,0}$ were determined by calibration measurement with R6G (D_{R6G}; 414 μm^2/s) for the green channel and Alexa647 ($D_{Alexa647}$;

330 μm^2/s) for the red channel.

$$w_{i,0} = \sqrt{4D_i \cdot \tau_{Di}} \qquad \text{- (22)}$$

$$s_i = \frac{z_{i,0}}{w_{i,0}} \qquad \text{- (23)}$$

where i indicates the green or red channel.

The effective observation volume of the green or red channels ($V_{i,\text{eff}}$) and the cross-correlation ($V_{C,\text{eff}}$) were calculated as follows:

$$V_{i,\text{ eff}} = \pi^{\frac{3}{2}} \cdot w^2{}_{i,0} \cdot z_{i,0} \qquad \text{- (24)}$$

$$V_{\text{C, eff}} = \left(\frac{\pi}{2}\right)^{\frac{3}{2}} \cdot (\omega^2{}_{\text{G},0} + \omega^2{}_{\text{R},0})(z^2{}_{\text{G},0} + z^2{}_{\text{R},0})^{\frac{1}{2}} \qquad \text{- (25)}$$

The amplitude of autocorrelation and cross-correlation functions is affected by the brightness in the case of a mixture of monomers and homodimers. However, this effect may be negligible, because the change in particle number was less than 5% of the total, and homodimeric fraction was also less than 7% of the total by our measurements (Fig. S18). Therefore, the average numbers of green fluorescent particles (N_G), red fluorescent particles (N_R), and particles showing the interaction between green and red fluorescent particles (N_C) are given by the following equations:

$$N_G = \frac{1}{G_G(0)-1} \quad \text{- (26)}$$

$$N_R = \frac{1}{G_R(0)-1} \quad \text{- (27)}$$

$$N_C = \frac{G_C(0)-1}{(G_G(0)-1)\cdot(G_R(0)-1)} \quad \text{- (28)}$$

The corrected number of fluorescent particles ($N_{G,corr}$ and $N_{R,corr}$) was calculated by removing the background effect from the measured number of fluorescent particles ($N_{G,meas}$ and $N_{R,meas}$), as follows[3,4]:

$$N_{i,corr} = \frac{N_{i,meas}\cdot(I_{i,meas}-I_{i,B})^2}{(I_{i,meas})^2} \quad \text{- (29)}$$

where i indicates the green or red channel (G or R), $N_{i,meas}$ is the average number of green or red fluorescent particles obtained from the autocorrelation functions and fitting analysis, $I_{i,meas}$ is the average intensity during FCCS measurement, and $I_{i,B}$ is the average background intensity obtained from the FCCS measurement of the non-transfected U2OS cell lysate. To use the corrected number of fluorescent particles from the green and red channels from equation (29), the apparent number from the cross-correlation amplitude was corrected as per the following equations:

$$N_{C,corr} = (G_{C,corr}(0)-1)\cdot N_{G,corr}\cdot N_{R,corr} \quad \text{- (30)}$$

where,

$$(G_{C,corr}(0)-1) = (G_{C,meas}(0)-1)\cdot\frac{I_{G,meas}\cdot I_{R,meas}}{\left(I_{G,meas}-I_{G,B}\right)\cdot\left(I_{R,meas}-I_{R,B}\right)} \quad \text{- (31)}$$

The concentration of each fluorescent protein was calculated using N_A (Avogadro's number) as given below:

$$\left[C_{i,\,\mathrm{corr}}\right]=\frac{N_{i,\,\mathrm{corr}}}{V_{i,\,\mathrm{eff}}\cdot \mathrm{N_A}} \qquad \text{- (32)}$$

where, i = G, R, or C (green channel, red channel, or cross-correlation)

The apparent dissociation constant for EGFP-GRs binding to Alexa647-labeled GREs was determined using the following equations:

$$K_{\mathrm{d,DNA,app}}=\frac{\left[G_{\mathrm{free}}\right]\cdot\left[R_{\mathrm{free}}\right]}{\left[Complex\right]} \qquad \text{- (33)}$$

where

$$\left[G_{\mathrm{free}}\right]=\left[C_{\mathrm{G,corr}}\right]-\left[C_{\mathrm{C,corr}}\right] \qquad \text{- (34)}$$

$$\left[R_{\mathrm{free}}\right]=\left[C_{\mathrm{R,corr}}\right]-\left[C_{\mathrm{C,corr}}\right] \qquad \text{- (35)}$$

$$\left[Complex\right]=\left[C_{\mathrm{C,corr}}\right] \qquad \text{- (36)}$$

The concentrations of the unbound EGFP-GRs or Alexa647-labeled GRE, [G_{free}] and [R_{free}] respectively, were calculated by subtracting the concentration of bound EGFP-GRs, [*Complex*], from the apparent EGFP and Alexa647-GREs concentrations, [$C_{\mathrm{G,corr}}$] and [$C_{\mathrm{R,corr}}$], respectively.

Electrophoretic mobility shift assay for DNA binding of EGFP-GRs to GREs

Following stimulation with 500 nM Dex for 20 min, transfected U2OS cells on a

10-cm cell culture dish were washed with ice-cold PBS (pH 7.4), trypsinized, and collected in PBS (pH 7.4) containing a trypsin inhibitor (4-[2-aminoethyl] benzenesulfonyl fluoride hydrochloride; Sigma-Aldrich). The number of cells was determined and an appropriate volume of lysis buffer (113 pL) was calculated based on the cell number and microwell volume to prepare EGFP or EGFP-GRs lysate with the same endogenous protein concentration as that extracted from a single cell in the microwell. After centrifugation (260 ×*g*, 2 min), the cell pellet was suspended in the calculated volume of lysis buffer and the cell lysates were sonicated on ice. The homogenates were then centrifugated (17400 ×*g*, 4 °C) for 15 min and the supernatants were collected. Equal total protein concentrations were confirmed in the EGFP and EGFP-GRs lysates.

The mixture of lysates and Alexa647-labeled GREs, EGFP-GR/WT lysate, and Alexa647-labeled PpGRE were electrophoresed in a 5% polyacrylamide gel with Tris-Acetate-EDTA (TAE) buffer at 4 °C[5]. The fluorescence of Alexa647 and EGFP was detected using a Typhoon Trio+ Variable Mode Imager (GE Healthcare).

Western blotting analysis

To investigate EGFP-GR complex formation, cell lysates were prepared as

described above. To separate the soluble and aggregate fractions, ultra-centrifugation was performed at 110000 *g* for 30 min at 4 °C. After collection of the supernatants, the pellet fraction was suspended in 15 μL of 1× Laemmli sample buffer. The 12 μL of supernatants were solubilized using 3 μL of 5× Laemmli sample buffer. The pellets and supernatants in Laemmli sample buffer were heat denatured at 95 °C for 5 min, electrophoresed in a 7.5% polyacrylamide gel, and then transferred onto a PVDF membrane (Bio-Rad Laboratories, Hercules, CA). The membranes were blocked in 5% skim milk for 1 h and washed three times in PBST buffer (137 mM NaCl, 2.7 mM KCl, 10 mM Na_2HPO_4, 2 mM KH_2PO_4, pH 7.4, 0.05% Tween 20) at room temperature. The membrane was washed and then incubated with primary monoclonal antibodies for GFP and GF200 (Nacalai Tesque; 1:1000 dilution in Can Get Signal Solution 1; Toyobo) overnight at 4 °C. After three washes with PBST, the membrane was incubated with a horseradish peroxidase (HRP)-conjugated goat anti-mouse IgG antibody (Santa Cruz Biotechnology; 1:1000 dilution in Can Get Signal Solution 2; Toyobo) for 1 h at room temperature. Then, the membrane was washed three times with PBST and subjected to reaction with ECL^{TM} Western Blotting Detection Reagents (GE Healthcare). Luminescence was detected using a LAS4000 mini biomolecular imager (Fujifilm, Japan).

To determine the relative expression level of EGFP-GR against endogenous GR in the U2OS cells, transfected U2OS cells on a 6-well plate were washed with ice-cold PBS (pH 7.4), trypsinized, and collected in PBS (pH 7.4). After centrifugation (260 ×*g*, 2 min), the cell pellet was suspended in the calculated volume of lysis buffer and the cell lysates were sonicated on ice. The homogenates were then centrifugated (17400 ×*g*, 4 °C) for 15 min and the supernatants were collected. Equal total protein concentrations were confirmed in the EGFP and EGFP-GRs lysates. The 12 μL of supernatants were solubilized using 3 μL of 5× Laemmli sample buffer. The supernatants in Laemmli sample buffer were heat denatured at 95 °C for 5 min, electrophoresed in a 7.5% polyacrylamide gel, and then transferred onto a PVDF membrane (Bio-Rad Laboratories, Hercules, CA). The membranes were blocked in 5% skim milk for 1 h and washed three times in PBST buffer (137 mM NaCl, 2.7 mM KCl, 10 mM Na_2HPO_4, 2 mM KH_2PO_4, pH 7.4, 0.05% Tween 20) at room temperature. The membrane was washed and then incubated with primary rabbit antibody for GR, Anti-GR (PC-170-200UL, Calbiochem (this product (PA1-510A) is currently available through Thermo Fisher Scientific); 1:1000 dilution in Can Get Signal Solution 1; Toyobo) overnight at 4 °C. After three washes with PBST, the membrane was incubated with a horseradish peroxidase (HRP)-conjugated goat anti-rabbit IgG antibody (111-035-144, Jackson

ImmunoResearch; 1:1000 dilution in Can Get Signal Solution 2; Toyobo) for 1 h at room temperature. Then, the membrane was washed three times with PBST and subjected to reaction with ECL™ Western Blotting Detection Reagents (GE Healthcare). Luminescence was detected using a LAS4000 mini biomolecular imager (Fujifilm, Japan).

Simulation analysis of a GR-GRE binding model using a finite-difference method

The left panel in Fig. S14 shows the interaction model between GR and GRE and their rate constants. The rate equation corresponding to the model can be expressed as follows:

$$\frac{dA}{dt} = -k_{AC}AE - 2k_{AB}A^2 - k_{CA}AC + 2k_{BA}B + k_{CA}C + k_{DC}D, \quad \text{- (37)}$$

$$\frac{dB}{dt} = -k_{BA}B - k_{BD}BE + k_{AB}a^2 + k_{DB}D, \quad \text{- (38)}$$

$$\frac{dC}{dt} = -k_{CA}C - k_{CD}AC + k_{AC}AE + k_{DC}D, \quad \text{- (39)}$$

$$\frac{dD}{dt} = -k_{DB}D - k_{DC}D + k_{BD}BE + k_{CD}AC, \quad \text{- (40)}$$

$$\frac{dE}{dt} = -k_{AC}AE - k_{BD}BE + k_{CA}C + k_{BD}D, \quad \text{- (41)}$$

where $A(t)$, $B(t)$, $C(t)$, $D(t)$, and $E(t)$ are the concentrations of monomer GR (mGR), homodimer GR (dGR), GRE-bound monomer GR (mGR-GRE), GRE-bound homodimer

GR (dGR-GRE), and free GRE (GRE), respectively. The parameters k_{xx} represent rate constants, with the direction denoted in the subscript. The simulation was performed based on a finite-difference method with a time resolution of 10 ns. Dissociation constants $K_{d,1}$, $K_{d,2}$, $K_{d,3}$, and $K_{d,4}$ [M] were determined, and the rate constants $1/k_{BA}$, $1/k_{CA}$, $1/k_{BD}$, and $1/k_{DC}$ were fixed as 10^{-5} [s]. The rate constants k_{AB}, k_{AC}, k_{BD}, and k_{CD} [$s^{-1}M^{-1}$] were calculated by

$$k_{AB} = \frac{k_{BA}}{K_{d,1}}, \quad - (42)$$

$$k_{AC} = \frac{k_{CA}}{K_{d,2}}, \quad - (43)$$

$$k_{BD} = \frac{k_{DB}}{K_{d,3}}, \quad - (44)$$

$$k_{CD} = \frac{k_{DC}}{K_{d,4}}. \quad - (45)$$

The initial concentration $A(0)$ was 1-100 nM, and $E(0)$ was 40 nM. The other initial concentrations were 0 nM. The concentrations of each species at the equilibrium state were obtained from concentrations at $t = 0.1$ ms. Finally, scatterplots showing the product of free concentrations of GR and GRE (([mGR]+[dGR])×[GRE]) against the complex concentration ([mGR-GRE]+[dGR-GRE]) were made using the equilibrium concentrations of each species.

Supplementary note

Maximum concentration of endogenous GR in the cell

The maximum concentration of endogenous GR has been estimated as 16200 fmol/mg in a cytotrophoblast using ligand saturation binding assay[6]. On the other hand, it has also been reported that COS-1 cells transfected with wild type GR contain 1420 fmol/mg and 111000 GR molecules/cell[7]. Using the expression level of transfected COS-1 cell as a calculation standard, the GR expression level in the cytotrophoblast (16200 fmol/mg) was calculated as 1266338 molecules/cell. Assuming 3 pL as the average cell volume[3], 1266338 molecules/cell was converted to 701 nM in the cytotrophoblast.

Total EGFP-GR microwell concentrations over 50 nM, which corresponds to 1.8 μM in the cell, seemed to reflect overexpression of GR by the living cell. Therefore, the fitting analysis was performed in the 0-50 nM concentration range to determine the $K_{d,homo}$.

Supplementary figures

Figure S1

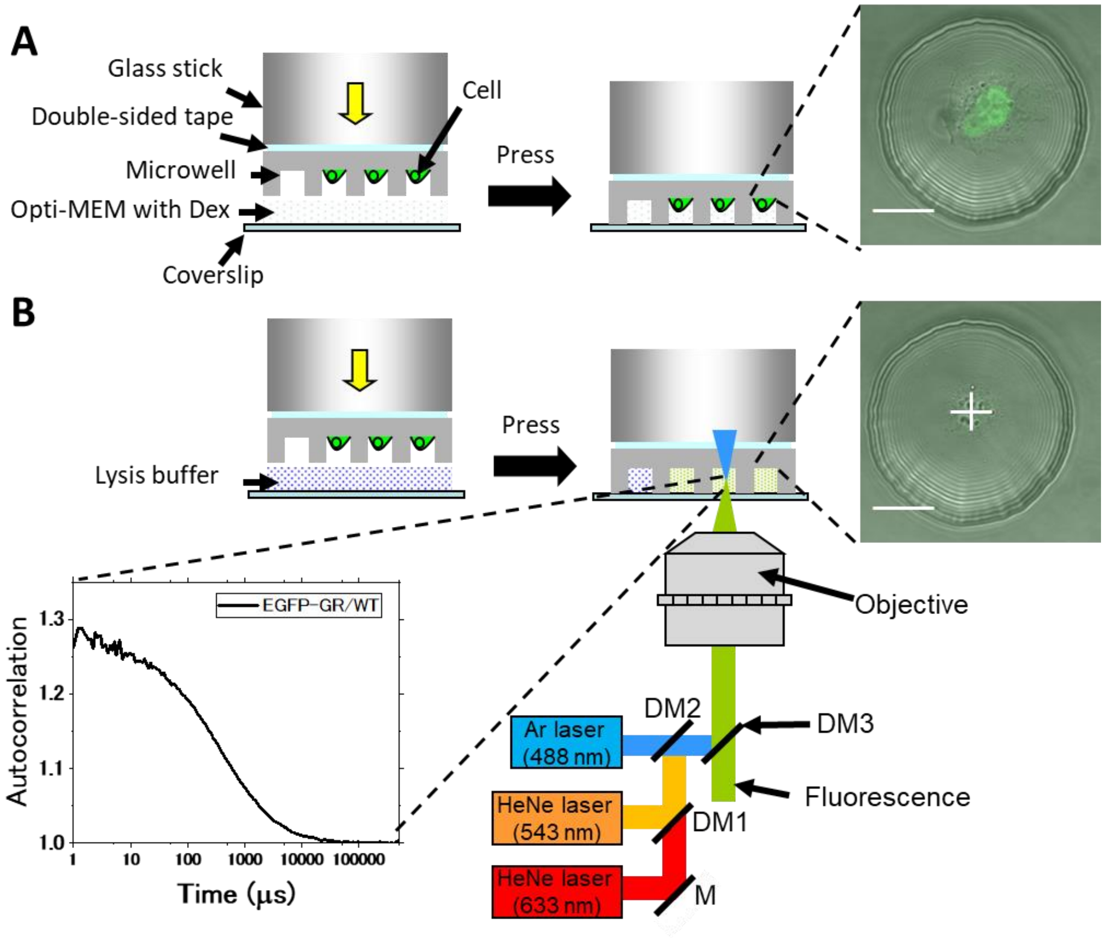

Figure S1. A method combining fluorescence correlation spectroscopy and a microwell system

(A) Single-cell method before cell lysis. A microwell containing a single cell was identified and noted. (Scale bar: 20 µm) **(B)** Single-cell method for cell lysis and triple-color FCS (tcFCS) measurement. Single cells were isolated into microwells, lysed, and tcFCS measurement was performed in the isolated cell lysate at the position represented by a white cross in the image. (Scale bar: 20 µm) DM: dichroic mirror, M: mirror.

Figure S2

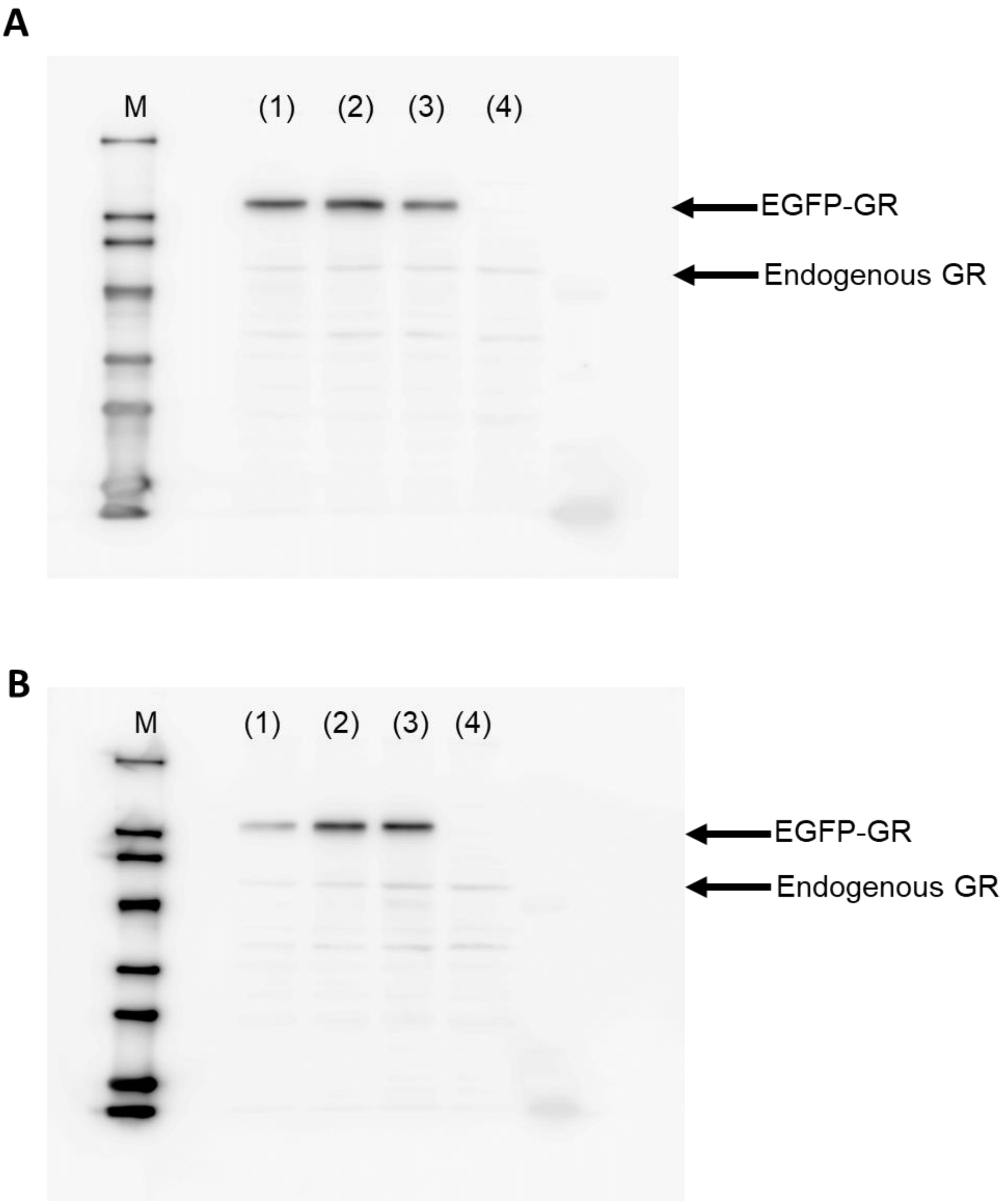

Figure S2. Relative expression levels of endogenous GR and EGFP-GR in U2OS cells

Western blotting was conducted with anti-GR antibody using lysates obtained from **(A)**

the experiments investigating the dissociation constant of GR homodimerization (Figs. 1 and 2) and **(B)** the transcriptional activity (Fig. 4). M: marker, (1): EGFP-GR/WT, (2): EGFP-GR/C421G, (3) EGFP-GR/A458T, (4) non-transfection. The expression of EGFP-GR is higher than that of endogenous GR, suggesting a relatively minimal effect of endogenous GR on determining the dissociation constant of EGFP-GR homodimerization and on the transcriptional activity assay.

Figure S3

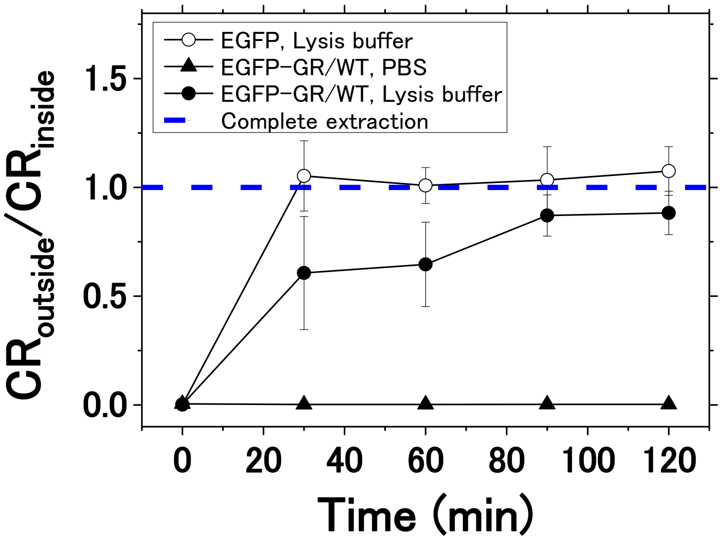

Figure S3. Extraction efficiency of EGFP-GR from single cells in a microwell

After cell lysis, the fluorescence intensity (CR: count rate) was estimated within and outside of each individual cell in a microwell. When EGFP-GR is completely extracted from a single cell, the fluorescence intensities in the inside and outside of a single cell are equal ($CR_{outside}/CR_{inside}$ = 1.0), as for EGFP. Black open circles: EGFP extracted by lysis buffer, black solid circles: EGFP-GR/WT extracted by lysis buffer, black solid triangles: EGFP-GR/WT extracted with phosphate buffered saline as a negative control, blue dashed line: complete extraction of EGFP-tagged proteins. Average and SD values were calculated from the data obtained from 15 individual cells.

Figure S4

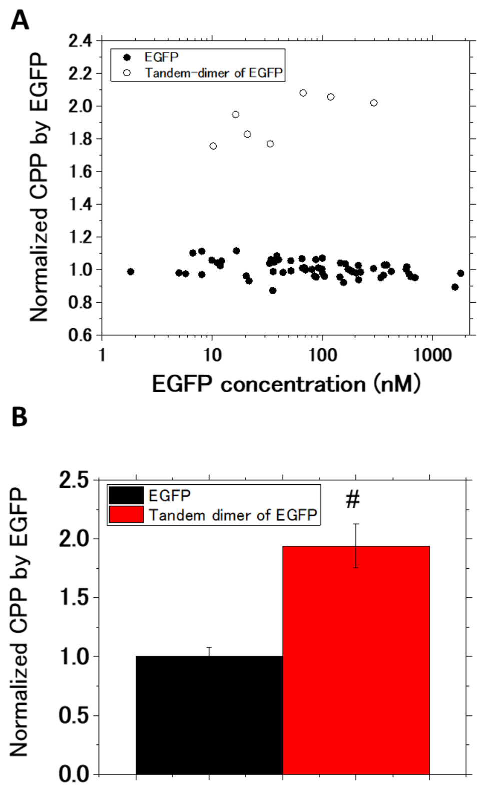

Figure S4 A comparison of counts per particle (CPP) of EGFP and tandem-dimer of EGFP

The counts per particle of EGFP and tandem-dimer of EGFP were measured by FCS in microwell. **(A)** The CPP of EGFP and the tandem-dimer of EGFP was normalized by the average CPP of EGFP in every experiment. Black open circles: tandem-dimer of EGFP (N = 7), black solid circles: (N = 61) **(B)** Average normalized CPP of EGFP and tandem-dimer of EGFP. The bars show the average value ± SD. Black bar: EGFP, red bar: tandem-dimer of EGFP. The CPP of tandem-dimer of EGFP was twice as high as that of EGFP,

suggesting that the CPP of EGFP-GR increases to double that of EGFP when all EGFP-GR molecules form homodimers. Statistical analysis was performed by Student's *t*-test. ($^{\#}$ $p = 1 \times 10^{-6}$)

Figure S5

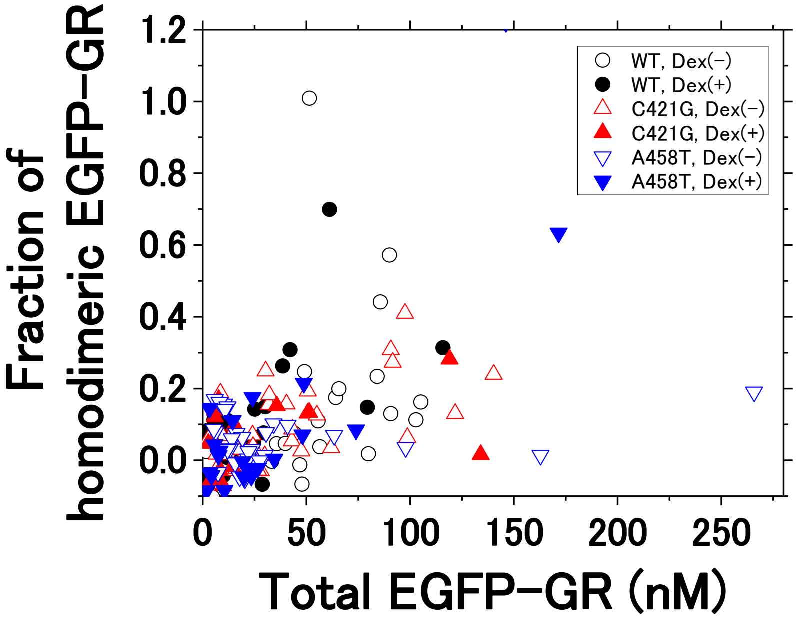

Figure S5 Fraction of homodimeric EGFP-GR

The fraction of homodimeric EGFP-GR was calculated from the normalized CPP (Figs. 1(d), 2(d), S6A, S6C and S6D). Black open circles: untreated wild type (WT) (N = 28), black solid circles: WT treated with Dex (N = 25), red open triangles: untreated C421G mutant (N = 35), red solid triangles: C421G mutant treated with Dex (N = 14), blue open triangles: untreated A458T mutant (N = 28), blue solid triangles: A458T mutant treated with Dex (N = 21).

Figure S6

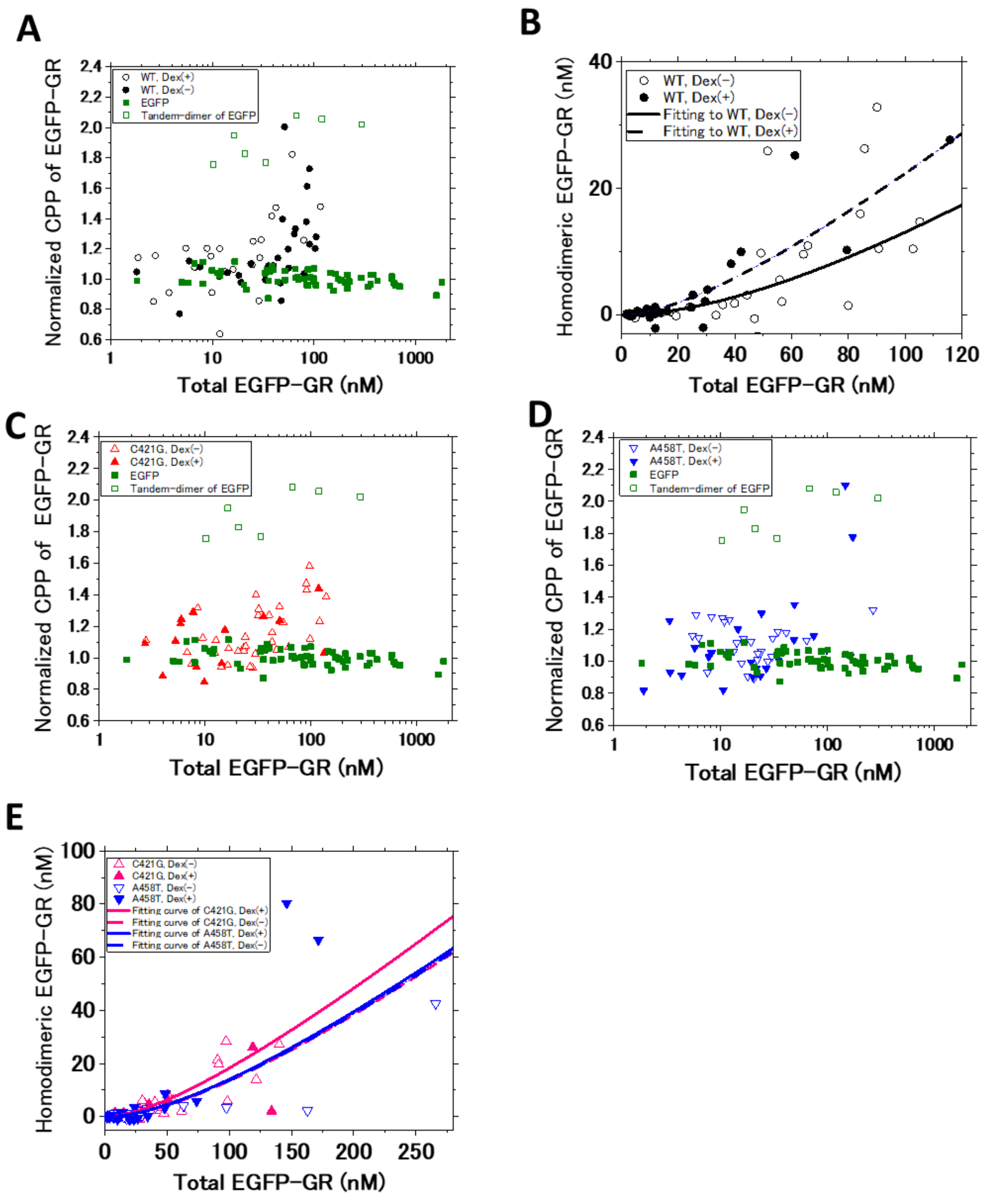

Figure S6. Whole range graphs of the normalized CPP and concentration of homodimeric EGFP-GR against the total concentration of EGFP-GR

(A, C and D) The normalized CPP WT EGFP-GR and GR mutants with and without Dex treatment. **(B, E)** The concentration of homodimeric EGFP-GR for WT GR and the GR

mutants with and without Dex treatment. Black open circles: untreated wild type (WT) (N = 28), black filled circles: WT treated with Dex (N = 25), red open triangles: untreated C421G mutant (N = 35), red filled triangles: C421G mutant treated with Dex (N = 14), blue open triangles: untreated A458T mutant (N = 28), blue filled triangles: A458T mutant treated with Dex (N = 21), green open squares: Tandem-dimer of EGFP (N = 7), green filled squares: EGFP (N = 61). Black dashed and solid lines: line of best fit of untreated WT and WT treated with Dex, red dashed and solid lines: line of best fit of untreated C421G mutant and C421G mutant treated with Dex, blue dashed and solid lines: line of best fit of untreated A458T mutant and A458T mutant treated with Dex.

Figure S7

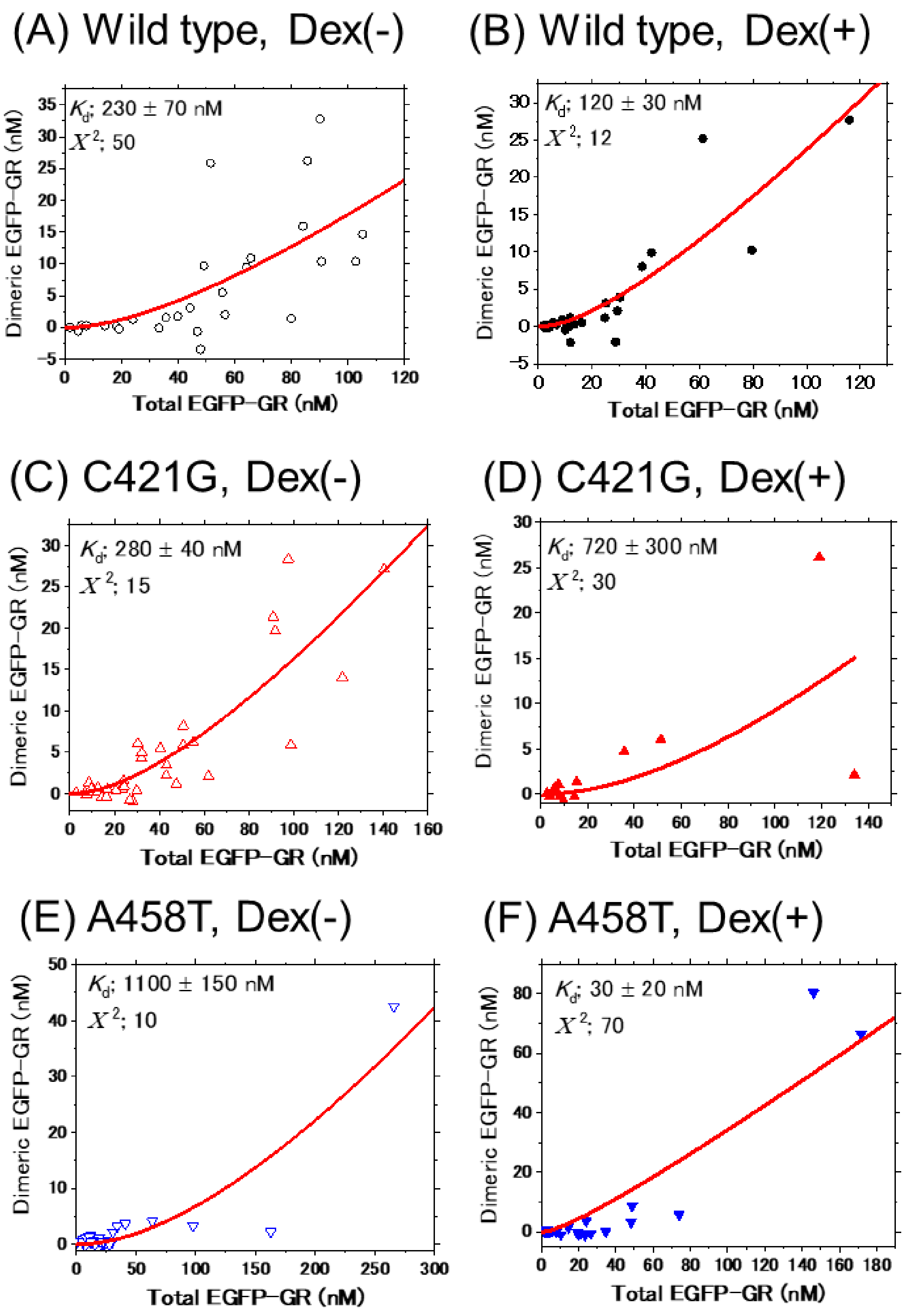

Figure S7. Determination of the dissociation constant of GR homodimerization by non-linear least squares fitting for the total range of concentrations

(A and B) Determination of the dissociation constant of GR homodimerization ($K_{d,homo}$) for wild type GR (WT) untreated ($K_{d,homo}$; 230 ± 70 nM, X^2; 50) and treated with Dex ($K_{d,homo}$; 120 ± 30 nM, X^2; 12). **(C and D)** Determination of the dissociation constant of GR homodimerization ($K_{d,homo}$) for the C421G mutant untreated ($K_{d,homo}$; 280 ± 40 nM, X^2; 15) and treated with Dex ($K_{d,homo}$; 720 ± 300 nM, X^2; 30). **(E and F)** Determination of the dissociation constant of GR homodimerization ($K_{d,homo}$) for the A458T mutant untreated ($K_{d,homo}$; 1100 ± 150 nM, X^2; 10) and treated with Dex ($K_{d,homo}$; 30 ± 20 nM, X^2; 70). X^2 represents the Chi-square value per degree of freedom.

Figure S8

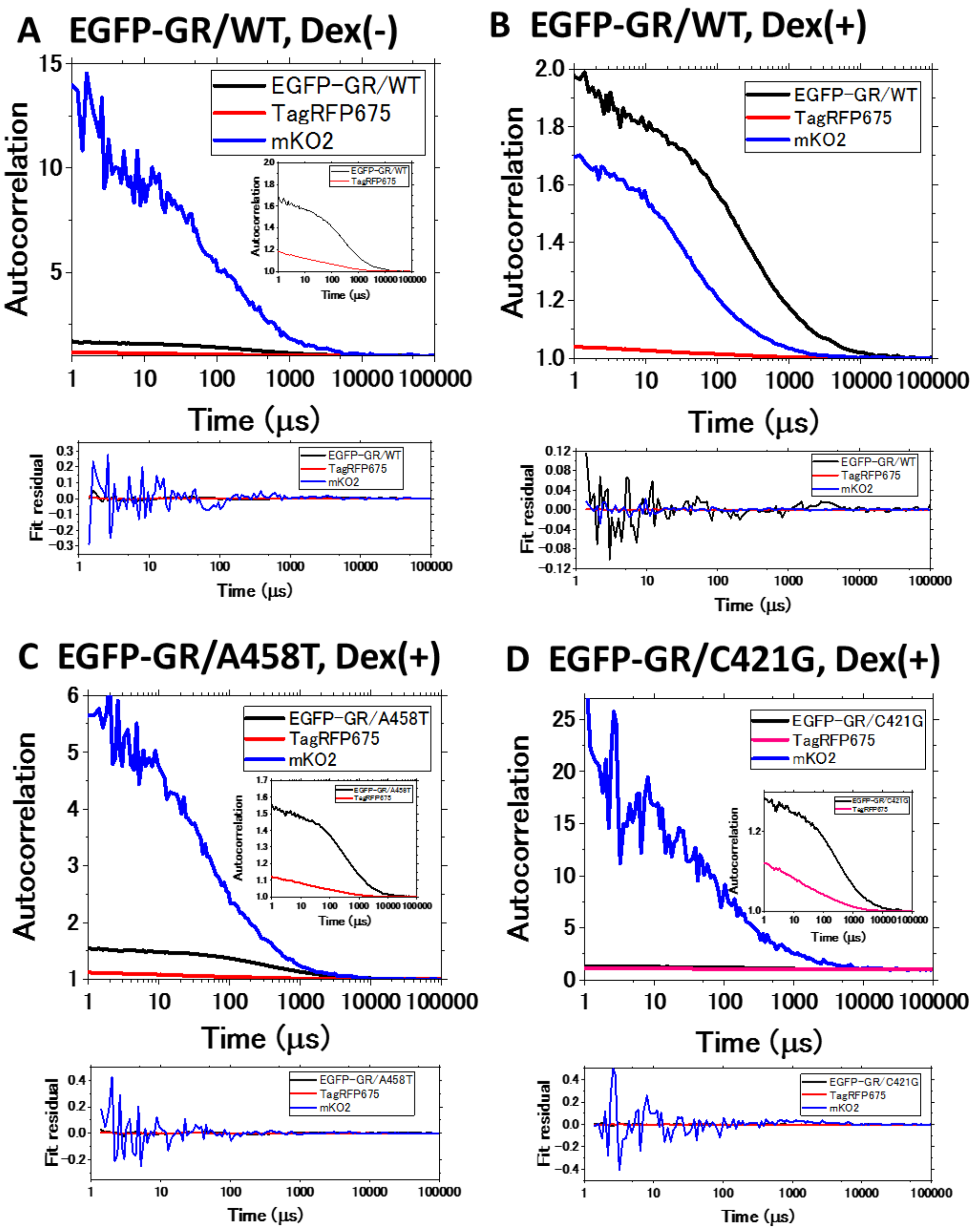

Figure S8. Typical autocorrelation functions and fit residuals of EGFP-GRs, mKO2, and TagRFP675 from a single cell

(A) Untreated EGFP-GR/WT, **(B)** EGFP-GR/WT treated with Dex, **(C)** EGFP-GR/A458T treated with Dex, **(D)** EGFP-GR/C421G treated with Dex. The insets show the enlarged graph at low autocorrelation amplitude. Black solid line: autocorrelation function of EGFP-GRs, red solid line: autocorrelation function of TagRFP675, blue solid line: autocorrelation function of mKO2.

Figure S9

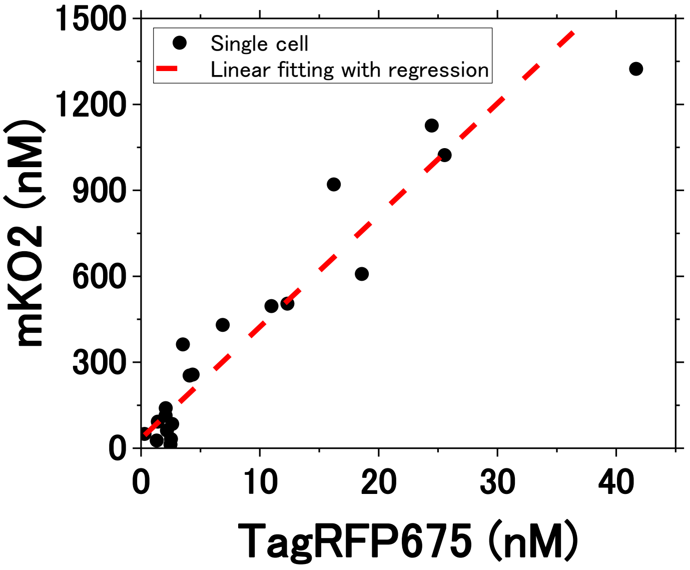

Figure S9. Confirmation of TagRFP675 as a transfection control

pCMV-TagRFP675 and pCMV-mKO2 were co-transfected into U2OS cells. The concentrations of TagRFP675 and mKO2 from single cells were determined using a tcFCS-microwell system. If a positive linear relationship between the concentrations of TagRFP675 and mKO2 is observed, the concentration of TagRFP675 can be used to determine the relative amount of transfected plasmid as a transfection control for the transcriptional activity assay.

Figure S10

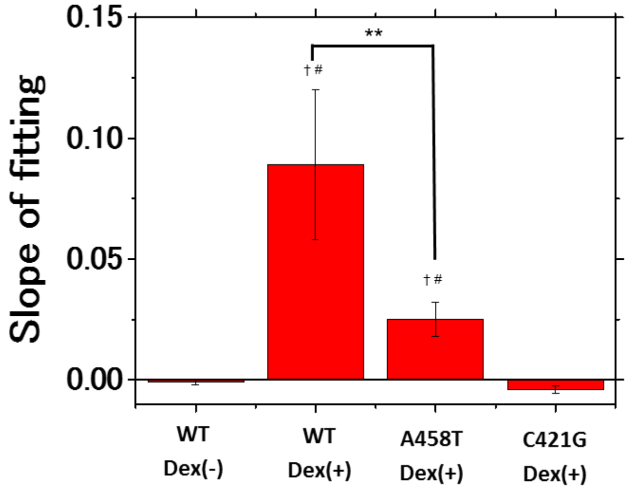

Figure S10. Significant differences were observed in the slopes of the lines of best fit representing the trend of concentration of GR homodimer versus transcriptional activity

The slopes of line of best fit were determined in Figs. 4(c) and 4(d). Bars show the average ± SE. Statistical analysis was performed by Student's *t*-test. (** $p<.01$, # $p<.01$ against WT without Dex treatment, † $p<.01$ against C421G mutant treated with Dex.)

Figure S11

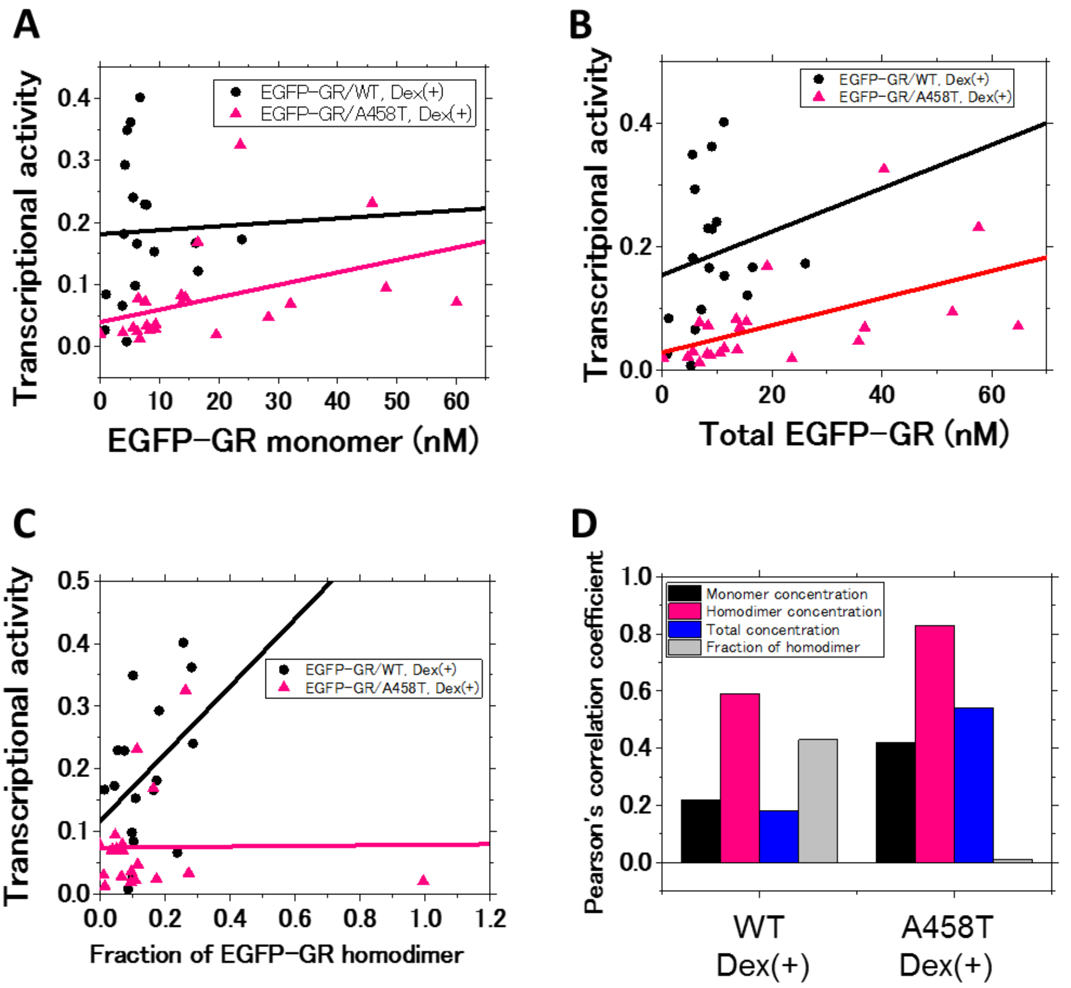

Figure S11 Pearson's correlation coefficient for transcriptional activity

(A-C) Lines of best fit via least squares of the transcriptional activity against **(A)** the concentration of monomer EGFP-GRs, **(B)** the total concentration of EGFP-GRs, and **(C)** the fraction of EGFP-GRs homodimer. Black filled circles: EGFP-GR/WT treated with Dex (N = 18), red filled triangles: EGFP-GR/A458T treated with Dex (N = 24), black and red solid line: line of best fit via least squares of EGFP-GR/WT and EGFP-GR/A458T

respectively. **(D)** Comparison of the Pearson's correlation coefficients for transcriptional activity determined by the linear least squares method in Figs. 4(c), 4(d) and S11A-C. Black bar: Pearson's correlation coefficient for monomer concentration, red bar: Pearson's correlation coefficient for homodimer concentration, blue bar: Pearson's correlation coefficient for total concentration, grey bar: Pearson's correlation coefficient for fraction of homodimer.

Figure S12

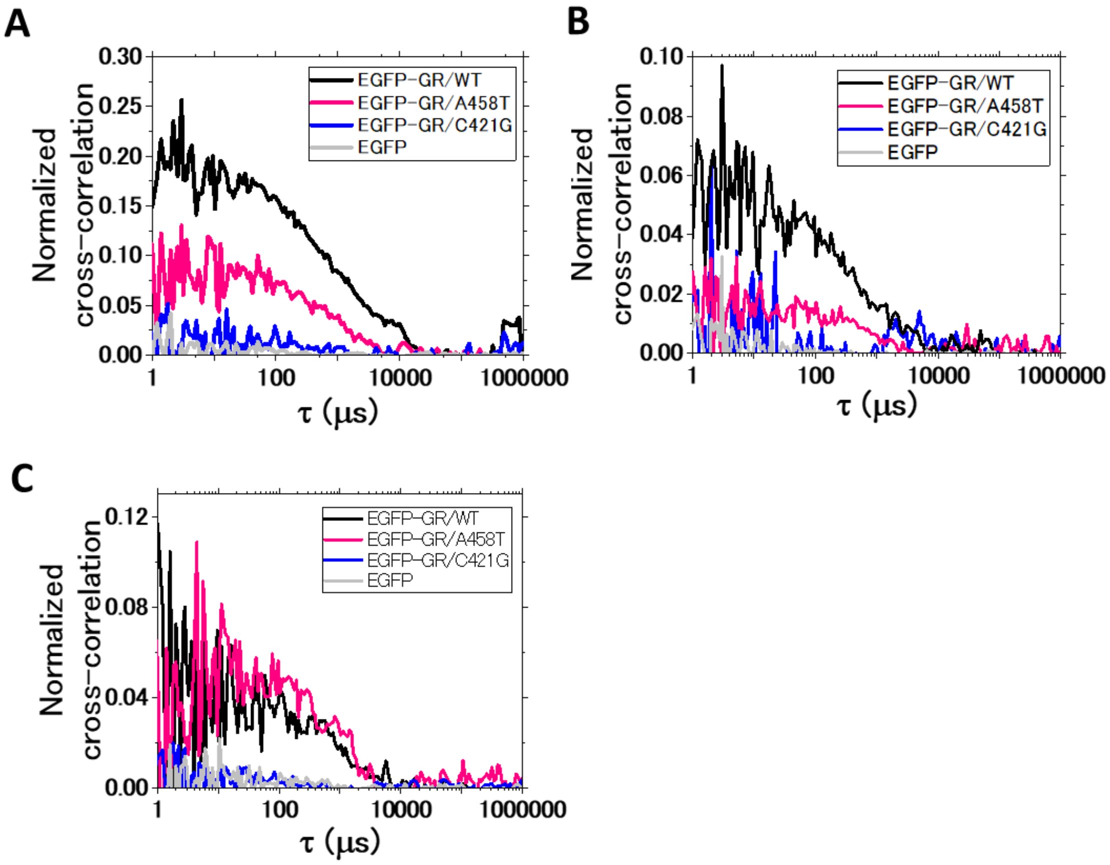

Figure S12. Typical normalized cross-correlation functions for the *in vitro* DNA binding analysis

Typical cross-correlation functions normalized by the autocorrelation amplitude of the red channel. Black line: EGFP-GR/WT, red line: EGFP-GR/A458T, blue line: EGFP-GR/C421G, grey line: EGFP. **(A)** PpGRE, **(B)** IpGRE, **(C)** hGRE.

Figure S13

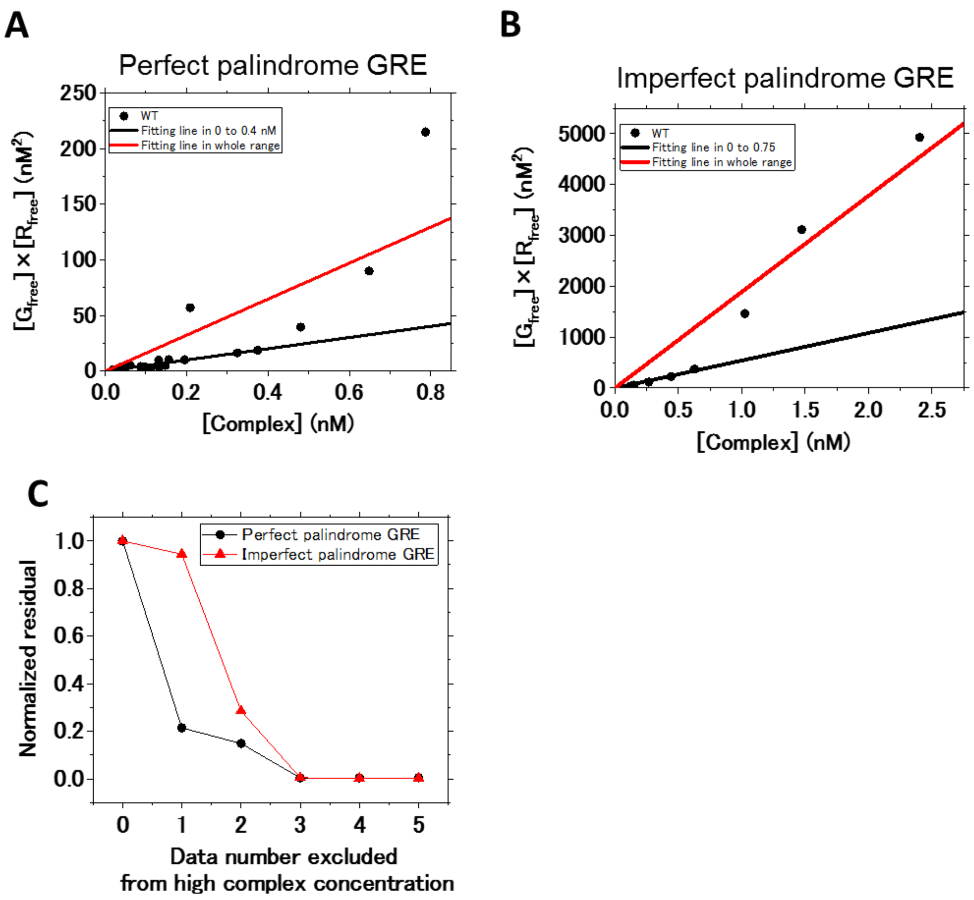

Figure S13. Lines of best fit via least squares over observed concentration range for the *in vitro* DNA binding analysis

(A, B) $K_{d,DNA,app}$ determination by linear least squares fitting in the mixture of EGFP-GR/WT and Alexa647-PpGRE or IpGRE. Black line: line of best fit, excluding three data points at a high concentration of complex, red line: line of best fit for all data. **(A)** PpGRE, **(B)** IpGRE. **(C)** Normalized fitting residual. The normalized fitting residual reached a plateau when three data points from the high complex concentration were excluded.

Figure S14

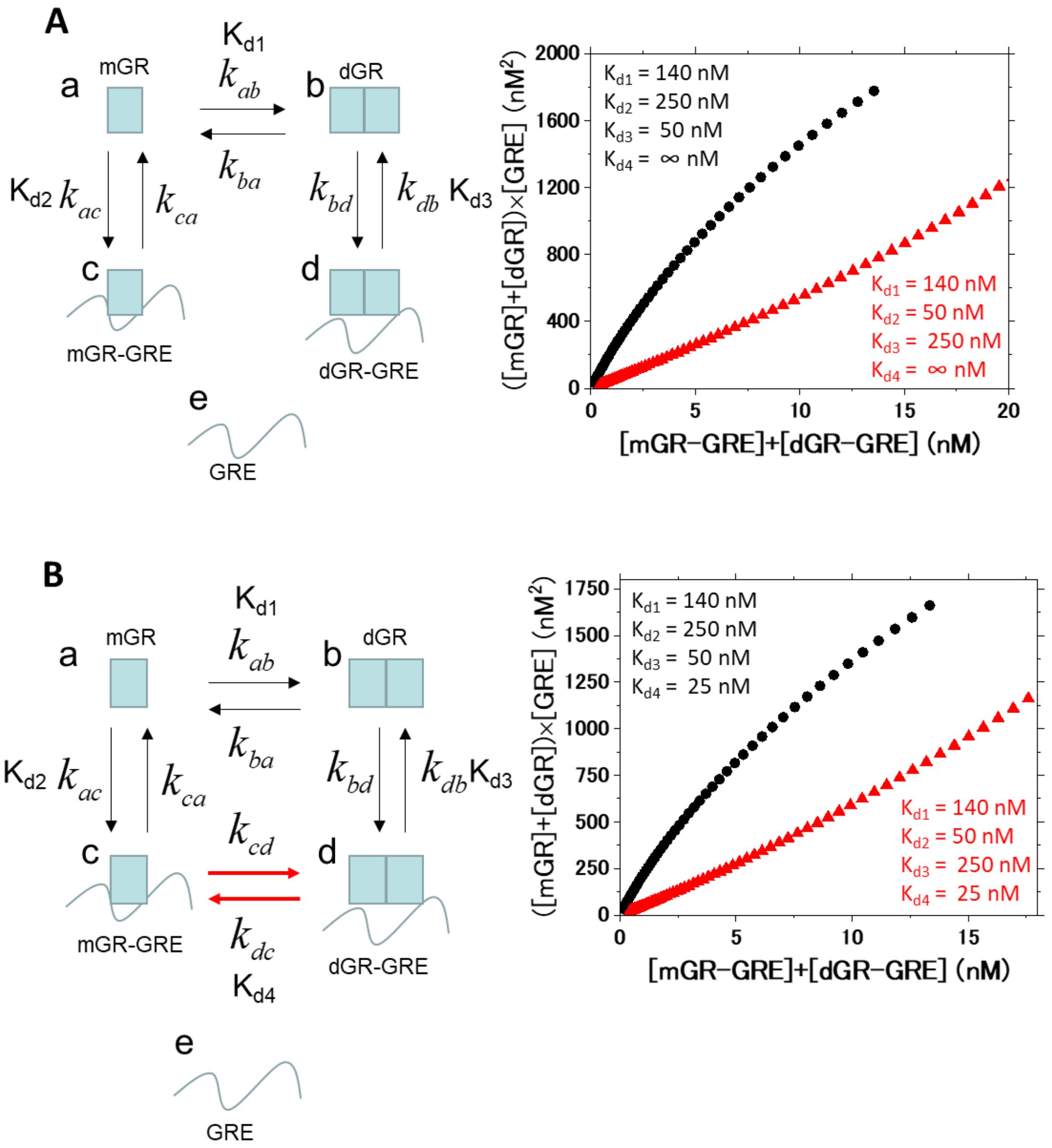

Figure S14. Simulation analysis for GR-GRE binding models

Simulation analysis was carried out by two models in the absence and presence of homodimerization on the GRE. Scatterplots were created representing the products of the free concentrations (([mGR]+[dGR])×[GRE]) against the complex concentration ([mGR-GRE]+[dGR-GRE]). **(A)** GR-GRE binding model in the absence of

homodimerization on the GRE. Black circles: simulation by K_{d1}= 140 nM, K_{d2}= 250 nM, K_{d3}= 50 nM, and K_{d4} = ∞ nM, red triangles: simulation by K_{d1}= 140 nM, K_{d2}= 50 nM, K_{d3}= 250 nM, and K_{d4} = ∞ nM. **(B)** GR-GRE binding model in the presence of homodimerization on the GRE (shown as red arrows in the model). Black circles: simulation by K_{d1}= 140 nM, K_{d2}= 250 nM, K_{d3}= 50 nM, and K_{d4} =25 nM, red triangles: simulation by K_{d1}= 140 nM, K_{d2}= 50 nM, K_{d3}= 250 nM, and K_{d4} =25 nM. K_{d1}, K_{d2}, K_{d3}, and K_{d4} represent dissociation constants for homodimerization in solution (a↔b), monomer binding (a↔c), homodimer binding (b↔d) and homodimerization on the GRE (c↔d), respectively.

Figure S15

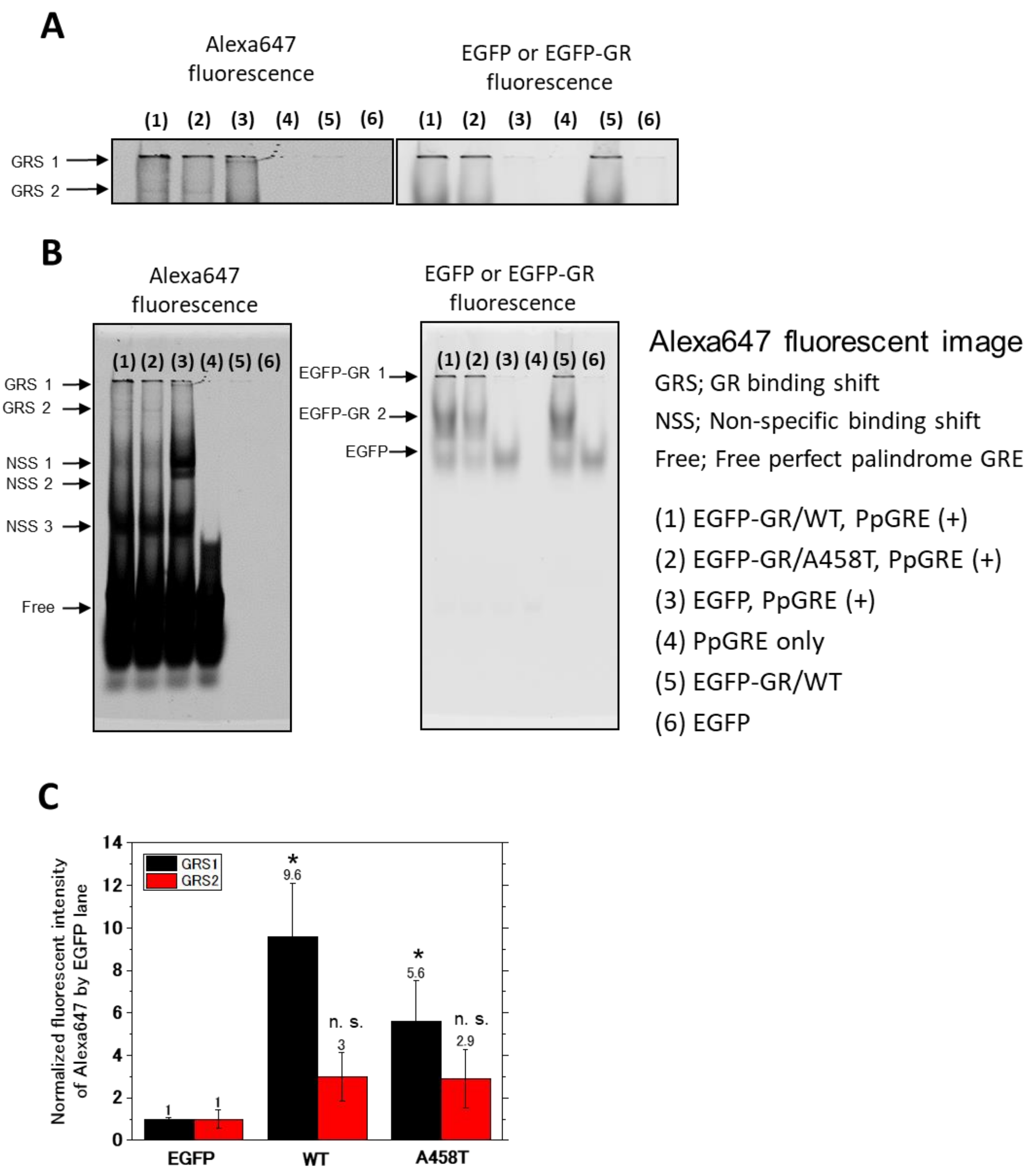

Figure S15. Electrophoretic mobility shift assay for the binding of EGFP-GRs to the perfect palindrome glucocorticoid response element (PpGRE)

(A) Cropped fluorescent images of GR shifts (GRS1 and GRS2). GRS1 shows higher fluorescent intensity in lanes 1 and 2 than the EGFP band in lane 3 (Fig. S15C). GRS2

was observed faintly in lanes 1 and 2, but fluorescent intensity was not significantly different (Fig. S15C). **(B)** Whole fluorescent images. The samples were loaded onto lanes of the gel as follows: (1) EGFP-GR/WT with PpGRE, (2) EGFP-GR/A458T with PpGRE, (3) EGFP with PpGRE, (4) PpGRE only, (5) EGFP-GR/WT only, (6) EGFP only. The shifts in the Alexa647 fluorescent image of the gel consist of two shifts for GR binding (GRS1 and GRS2) and three non-specific binding shifts (NSS1, NSS2 and NSS3). A band is also observed representing free Alexa647-labeled PpGRE. In the fluorescent image of EGFP or EGFP-GR, fluorescent intensity was observed at three positions: EGFP-GR1, EGFP-GR2, and EGFP. **(C)** Normalized fluorescent intensity of GRS1 and GRS2. The bars show average ± SD. Student's *t*-test was carried out against EGFP lane in GRS1 and GRS2 (* $p<.05$).

Figure S16

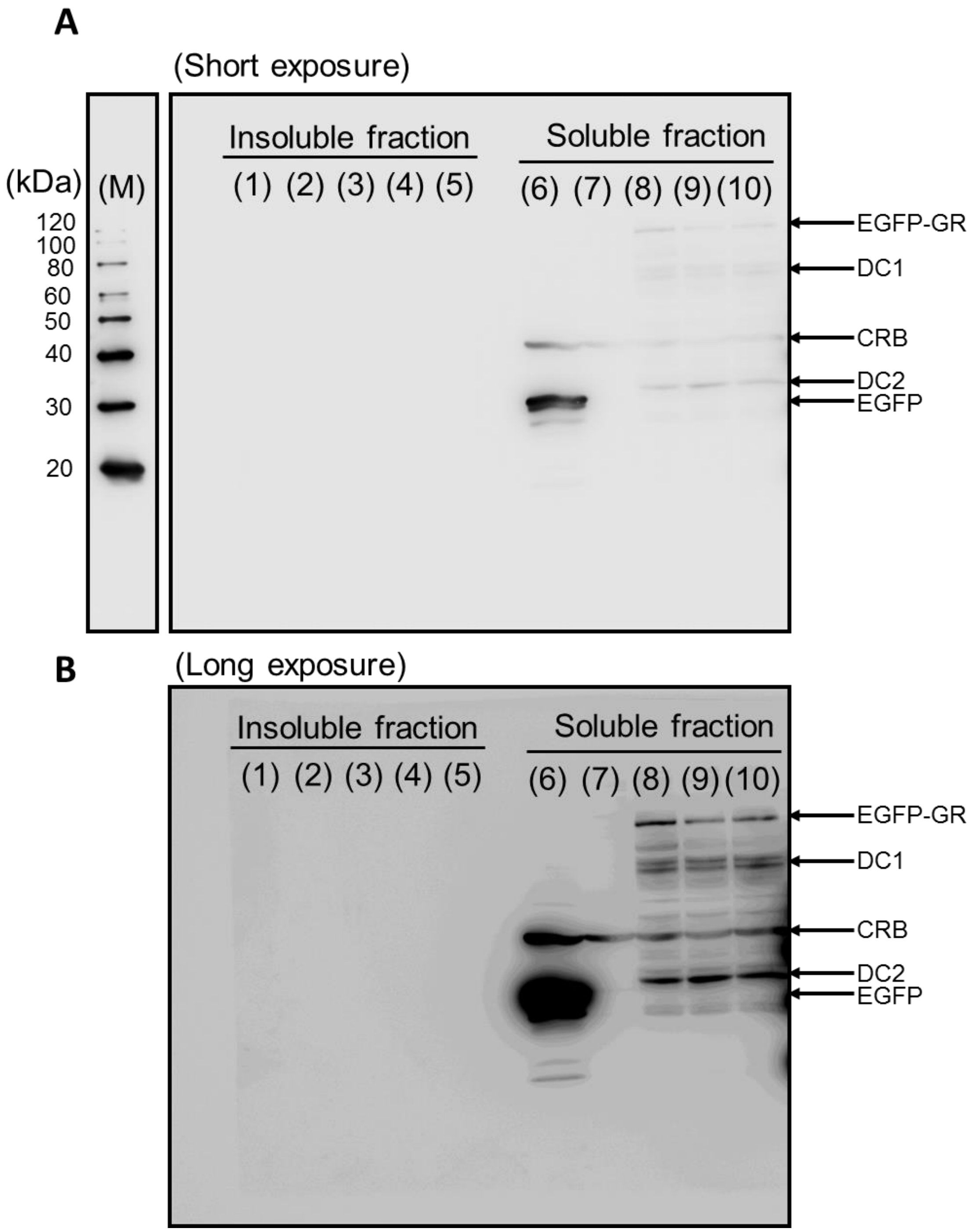

Figure S16. Western blotting analysis of soluble and insoluble (aggregate) fractions

The samples were loaded onto lanes of the gel as follows: the insoluble fractions of (1)

EGFP lysate, (2) non-transfected cell lysate, (3) EGFP-GR/C421G lysate, (4) EGFP-GR/A458T lysate, (5) EGFP-GR/WT lysate, and the soluble fraction of (6) EGFP lysate, (7) non-transfected cell lysate, (8) EGFP-GR/C421G lysate, (9) EGFP-GR/A458T lysate, and (10) EGFP-GR/WT lysate, (M) molecular marker (MagicMark^{TM} XP Western Protein Standard (Thermo Fisher Scientific)). Several bands representing EGFP-GR/WT and EGFP-GR/A458T were observed in the soluble fractions due to degradation components (DC: DC1 and DC2). However, the bands were not detected in the insoluble fractions, suggesting that aggregates of EGFP-GRs are not formed extensively. **(A)** image for short binning time, **(B)** image for long binning time.

Figure S17

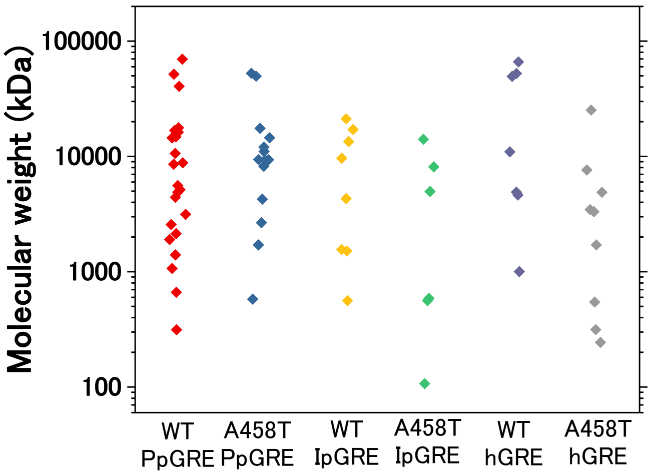

Figure S17. Molecular weight of DNA-GR complexes

The molecular weight of DNA-GR complexes was calculated by sphere model using diffusion constants from cross-correlation functions. Values largely ranged from 250 kDa to 70 MDa.

Figure S18

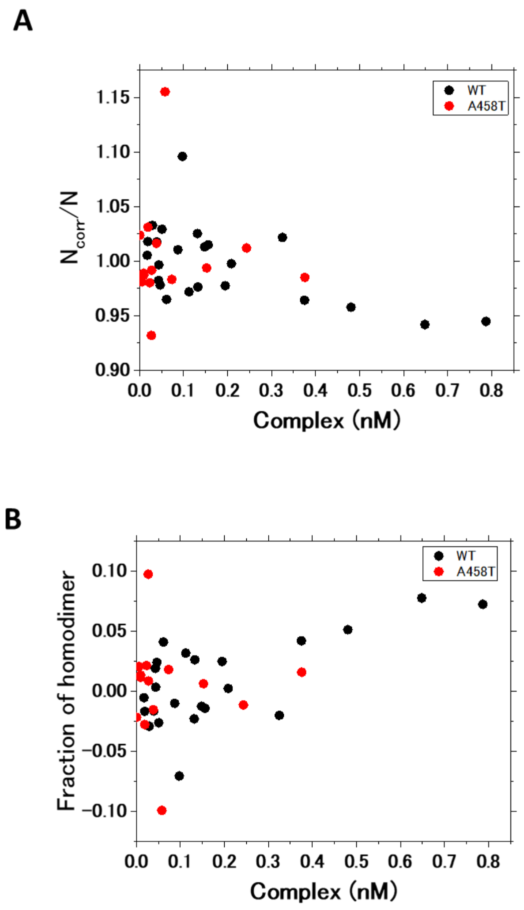

Figure S18 Calculation errors for particle numbers from FCCS measurements

If monomer and homodimer are present in the lysate, the number of particles estimated from FCS measurements is not inversely proportional to the amplitude of the autocorrelation functions. **(A)** Numbers of particles calculated either without (N) or with (N_{corr}) consideration for the brightness of monomer and homodimer. Almost all plots for the ratio of N_{corr} against N yielded values in the range of 0.95 to 1.05. **(B)** The fraction of homodimeric EGFP-GR was generally less than 0.07. Black circles: WT GR, red circles:

A458T mutant.

Table S1

Measured fluorescent molecule	Detection channel	Counts per particle (kHz) 543 nm laser excitation	Counts per particle (kHz) 488 nm and 633 nm excitation
EGFP	EGFP (BP 505-550)		10.1
	mKO2 (BP 565-615)	0.01	
	TagRFP675 (LP 650)		0.03
mKO2	EGFP (BP 505-550)		0.39
	mKO2 (BP 565-615)	8.26	
	TagRFP675 (LP 650)		0.40
TagRFP675	EGFP (BP 505-550)		0.13
	mKO2 (BP 565-615)	0.06	
	EGFP (LP 650)		6.84

Table S1. Absence of cross-talk in triple-color FCS measurement.

EGFP, mKO2, or TagRFP675 were measured by two optical setups for EGFP and TagRFP675, and for mKO2. To prevent cross-talk between channels, FCS measurements for EGFP and TagRFP675, and for mKO2 were performed sequentially. If cross-talk occurred between channels, a relatively higher counts per particle (CPP) value is observed in both the detection channel and cross-talk channel. Here, a high CPP was observed in the detection channel, but not in the cross-talk channel, suggesting that this method avoids the cross-talk in triple-color FCS measurement.

Supplementary References

1. Müller, C. B. *et al.* Precise measurement of diffusion by multi-color dual-focus fluorescence correlation spectroscopy. *EPL (Europhysics Lett.* **83,** 46001 (2008).
2. Kapusta, P. Absolute diffusion coefficients: compilation of reference data for FCS calibration. *Appl. note* 0–1 (2010).
3. Oasa, S., Sasaki, A., Yamamoto, J., Mikuni, S. & Kinjo, M. Homodimerization of glucocorticoid receptor from single cells investigated using fluorescence correlation spectroscopy and microwells. *FEBS Lett.* **589,** 2171–2178 (2015).
4. Thompson, N. L. in *Topics in Fluorescence Spectroscopy* 337–378 (Kluwer Academic Publishers, 2002). doi:10.1007/0-306-47057-8_6
5. Drouin, J. *et al.* Homodimer formation is rate-limiting for high affinity DNA binding by glucocorticoid receptor. *Mol. Endocrinol.* **6,** 1299–309 (1992).
6. Driver, P. M. *et al.* Expression of 11 beta-hydroxysteroid dehydrogenase isozymes and corticosteroid hormone receptors in primary cultures of human trophoblast and placental bed biopsies. *Mol. Hum. Reprod.* **7,** 357–63 (2001).
7. Robertson, S., Rohwer, J. M., Hapgood, J. P. & Louw, A. Impact of glucocorticoid receptor density on ligand-independent dimerization, cooperative ligand-binding and basal priming of transactivation: a cell culture model. *PLoS*

One **8,** e64831 (2013).
